# A novel strategy for an anti-idiotype vaccine: nanobody mimicking neutralization epitope of porcine circovirus type 2

**DOI:** 10.1128/jvi.01650-23

**Published:** 2024-01-25

**Authors:** Yingying Deng, Yamin Sheng, Guixi Zhang, Yani Sun, Lei Wang, Pinpin Ji, Jiahong Zhu, Gang Wang, Baoyuan Liu, En-Min Zhou, Xuehui Cai, Yabin Tu, Julian A. Hiscox, James P. Stewart, Yang Mu, Qin Zhao

**Affiliations:** 1 Department of Preventive Veterinary Medicine, College of Veterinary Medicine, Northwest A&F University, Yangling, Shannxi, China; 2 Engineering Research Center of Efficient New Vaccines for Animals, Universities of Shaanxi Province and Ministry of Education, Yangling, China; 3 Key Laboratory of Ruminant Disease Prevention and Control (West), Ministry of Agriculture and Rural Affairs, Yangling, China; 4 College of Veterinary Medicine, Shandong Agricultural University, Tai'an, China; 5 Harbin Veterinary Research Institute, Chinese Academy of Agricultural Sciences, Harbin, China; 6 Department of Infection Biology and Microbiomes, Institute of Infection, Veterinary and Ecological Sciences, University of Liverpool, Liverpool, United Kingdom; Lerner Research Institute, Cleveland Clinic, Cleveland, Ohio, USA

**Keywords:** anti-idiotype antibody, nanobody, epitope, PCV2, vaccine

## Abstract

Vaccination is the most effective method to protect humans and animals from diseases. Anti-idiotype vaccines are safer due to their absence of pathogens. However, the commercial production of traditional anti-idiotype vaccines using monoclonal and polyclonal antibodies (mAb and pAb) is complex and has a high failure rate. The present study designed a novel, simple, low-cost strategy for developing anti-idiotype vaccines with nanobody technology. We used porcine circovirus type 2 (PCV2) as a viral model, which can result in serious economic loss in the pig industry. The neutralizing mAb-1E7 (Ab1) against PCV2 capsid protein (PCV2-Cap) was immunized in the camel. And 12 nanobodies against mAb-1E7 were screened. Among them, Nb61 (Ab2) targeted the idiotype epitope of mAb-1E7 and blocked mAb-1E7’s binding to PCV2-Cap. Additionally, a high-dose Nb61 vaccination can also protect mice and pigs from PCV2 infection. Epitope mapping showed that mAb-1E7 recognized the ^75^NINDFL^80^ of PCV2-Cap and ^101^NYNDFLG^107^ of Nb61. Subsequently, the mAb-3G4 (Ab3) against Nb61 was produced and can neutralize PCV2 infection in the PK-15 cells. Structure analysis showed that the amino acids of mAb-1E7 and mAb-3G4 respective binding to PCV2-Cap and Nb61 were also similar on the amino acids sequences and spatial conformation. Collectively, our study first provided a strategy for producing nanobody-based anti-idiotype vaccines and identified that anti-idiotype nanobodies could mimic the antigen on amino acids and structures. Importantly, as more and more neutralization mAbs against different pathogens are prepared, anti-idiotype nanobody vaccines can be easily produced against the disease with our strategy, especially for dangerous pathogens.

**IMPORTANCE:**

Anti-idiotype vaccines utilize idiotype-anti-idiotype network theory, eliminating the need for external antigens as vaccine candidates. Especially for dangerous pathogens, they were safer because they did not contact the live pathogenic microorganisms. However, developing anti-idiotype vaccines with traditional monoclonal and polyclonal antibodies is complex and has a high failure rate. We present a novel, universal, simple, low-cost strategy for producing anti-idiotype vaccines with nanobody technology. Using a neutralization antibody against PCV2-Cap, a nanobody (Ab2) was successfully produced and could mimic the neutralizing epitope of PCV2-Cap. The nanobody can induce protective immune responses against PCV2 infection in mice and pigs. It highlighted that the anti-idiotype vaccine using nanobody has a very good application in the future, especially for dangerous pathogens.

## INTRODUCTION

Vaccination is considered one of the cheapest and most effective medical intervention methods to protect humans and animals from diseases (1). Through vaccination, the morbidity and mortality rates of tetanus, measles, polio, mumps, rubella, pneumococci, and hepatitis B have been reduced by 97%–99% (2, 3). Especially for COVID-19 as the global epidemic of acute respiratory disease in recent years, the vaccines saved nearly 20 million lives in the first year (4). So, many technologies have been established to develop the vaccines, and many kinds of commercial vaccines, including inactivation, live attenuated, subunits, peptides, live vectors, anti-idiotype, DNA, and mRNA, have been produced (5). Out of which, the anti-idiotype vaccine is immunoglobulin and has enhanced safety and scalability. Compared to other relatively mature vaccine technologies, only a few laboratory vaccines based on anti-idiotype antibodies have been described, and there are no commercial products to date (6).

As we know, the anti-idiotype vaccines were designed according to Jernes’ idiotype network theory. The theory proposes that the host immune system generates a cascade of antibodies interacting with each other (7). Specifically, after the hosts’ contact with the antigens, they can produce the antibodies against the antigen (Ab1), the antibodies against the variable region of Ab1 (also called an anti-idiotype antibody, Ab2), the antibodies against the variable region of Ab2 (Ab3) and in turn (8). The Ab2 can be divided into four types: Ab2α, Ab2β, Ab2γ, and Ab2δ. The Ab2β can mimic the antigen and be a surrogate antigen for developing anti-idiotype vaccines (9). Previously, some papers documented that the anti-idiotype vaccine is feasible and practical in the prevention and treatment of viral diseases (10, 11), bacterial diseases (12), and cancer (13). All anti-idiotype vaccines were designed and researched using mice to produce monoclonal antibodies (mAb) by traditional hybridoma technology. However, it is not easy to screen anti-idiotypic antibodies because the idiotype of an antibody exists in the hypervariable region (8). The recognition ability of anti-idiotypic antibodies should be considered, and the immune response in the non-idiotypic region should also be reduced (14). Additionally, the molecules of mAb are large, and their binding regions for antigens are composed of all six complementarity-determining regions (CDRs) of the heavy and light chains (15). When anti-idiotypic antibodies are produced in large quantities with the expressing systems *in vitro*, the variable regions of heavy and light chains need to be correctly expressed and assembled *in vitro* to maintain the activity of the original antibodies. So, the commercial production process of anti-idiotypic antibodies from mAb is complicated, and the failure rate is very high (16).

Recently, the single domain antibody (sdAb), also known as nanobodies, has been derived from the variable region of heavy chain antibody (VHH) in camelids (17). They have been used in many fields, including biological diagnostics, therapeutic drugs, protein function (18–22), and structure analysis (23). The molecular weight of the nanobody is small, only 12–15 kDa. Compared with traditional antibodies, nanobodies have many advantages, such as higher specificity, affinity, solubility, ability to bind antigens, low cost, and easy mass production (24). Nanobody can be screened by phage display technology, which is simple, rapid, and economical and avoids the problems of long cycles, high cost, and difficult genetic manipulation for production using hybridoma technology (25). So, nanobodies can serve as a new approach to developing anti-idiotypic vaccines. Then, based on the advantages of nanobody, we designed that traditional neutralizing mAb is used to immunize camels. Anti-idiotype nanobodies against the idiotype on the variable region of the neutralizing mAb were subsequently screened and identified to mimic the neutralizing epitope of an antigen. More importantly, anti-idiotype nanobodies can be produced *in vitro* on a large scale and at low production costs, making it a very promising market application.

Porcine circovirus type 2 (PCV2) is a non-enveloped, the smallest DNA animal virus with a diameter of approximately 17 nm (26–28). The viral open reading frame 2 (ORF2) encodes the capsid protein (Cap). The PCV2-Cap, a major immunogenic protein of PCV2, was a target for developing vaccines and serodiagnostic assays (29). Commercialized PCV2 vaccines are divided into inactivated and subunit vaccines that use Cap as the immunogen (30). The inactivated vaccine is commonly produced in PK-15 cells with low yield due to poor propagation of PCV2 in PK-15 cells (31). The baculovirus system was used to manufacture the capsid protein-based virus-like particle (VLP) vaccine, which requires expensive ultracentrifugation or chromatography for purification (32). So, the production of current PCV2 vaccines is time-consuming and expensive.

Meanwhile, PCV2 vaccines cannot provide complete protection, and viremia can be detected in some immunized pigs (30). In the present study, an anti-PCV2 Cap mAb was used as a template to produce the β-anti-idiotype nanobodies, which can mimic the neutralization epitope of PCV2-Cap in structure and function. We demonstrated that immunization with β-anti-idiotype nanobodies can induce Ab3 (Ab1′) in pigs and mice and protect against PCV2 infection. More importantly, the platforms and technologies proposed in this study have no restrictions on the selection of antibodies and have the possibility of a broad-scope application. Up to now, more and more neutralizing mAbs have been identified and characterized, particularly those that bind to the conformational epitope(s) of antigen, and this platform can be used to prepare anti-idiotype nanobody vaccines with these neutralizing mAbs.

## RESULTS

### MAb-1E7 acts as a neutralizing antibody against PCV2b infection

**Fig 1:**
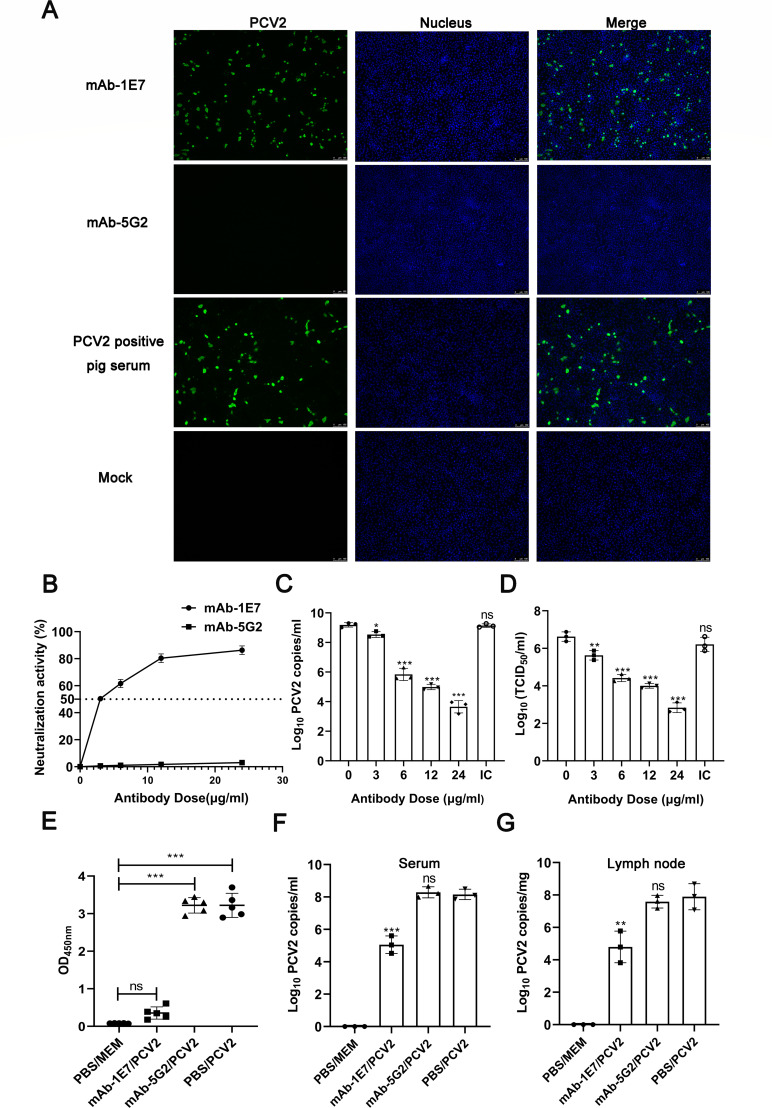
The mAb-1E7 neutralized PCV2b infection in PK-15 cells and blocked PCV2b infection in mice. (**A**) Detection of mAb-1E7 binding to PCV2b infected PK-15 cells by IFA. (**B**) Neutralization activity of mAb-1E7 with different concentrations. mAb-5G2 was as IgG1 isotype control (IC). A mean neutralizing activity of >50% was considered to represent neutralization. The minimum antibody concentration that reduced the number of PCV2-infected cells by 50% was defined as the IC50 for the antibody. The dotted line indicates the cutoff value. Error bars indicate the standard deviations. (**C**) PCV2 DNA copies in infected cells detected by quantitative real-time PCR (qPCR). PK-15 cells infected with PCV2b SH strain (100 TCID50) pretreated with mAb-1E7 at indicated concentrations. mAb-5G2 was as IgG1 IC. (**D**) Virus titers from the cell culture supernatant by TCID50 detection. mAb-5G2 was as IgG1 isotype control (IC). (**E**) Serum samples from mice were collected at 28 dpi and determined using indirect ELISA to detect anti-PCV2 antibodies. (**F**) PCV2b DNA copies in the sera from the mice at 28 dpi were measured by qPCR. (**G**) Quantification of PCV2b DNA copies in lymph nodes of mice by qPCR. Lymph nodes were collected from the mice at 28 dpi. Data are expressed as the mean ± standard deviation of three repeats. Significant differences between groups were compared and marked by *P < 0.05, **P < 0.01, and ***P < 0.001.

The mAb-1E7 was produced by the hybridoma technology and was kindly provided by Prof. Cai at Harbin Veterinary Research Institute, Chinese Academy of Agricultural Sciences. The antigenicity and neutralization of mAb-1E7 were determined in the present study. First, the reaction between mAb-1E7 and PCV2-Cap was analyzed with immunoassays. Soluble expressed and purified recombinant PCV2-Cap was also kindly presented by Pro. Cai (33). Using the purified PCV2-Cap as coated antigen, the indirect ELISA showed that the mAb-1E7 could specifically bind to the PCV2-Cap (Fig. S1A). Western blot also showed the same results as indirect ELISA (Fig. S1B), suggesting that the mAb-1E7 may recognize a linear epitope. Additionally, after PK-15 cells were inoculated with 100 TCID_50_ PCV2b strain SH, immunofluorescence assay (IFA) showed that the mAb-1E7 could also recognize the Cap in the PCV2-infected cells, whereas the mAb did not react with uninfected PK-15 cells (Fig. 1A). Moreover, serum containing polyclonal antibodies from PCV2-infected pigs was used as a positive control to confirm the replication of PCV2 in PK-15 cells.

Second, the neutralization of mAb-1E7 was determined *in vitro*. We first measured the 50% inhibitory concentration (IC_50_) values of mAb-1E7 using virus neutralization assays. PCV2b strain SH (200 TCID_50_, 100  µL) was incubated with 100 µL of serially diluted mAb-1E7 for 1 h at 37°C. Then, the mixtures were separately added into PK-15 cells. At 48 hours post inoculation (hpi), the IFA was performed to quantify viral infection. And then, IC_50_ were calculated. The results showed that the IC_50_ was 3.124 µg/mL (Fig. 1B). Compared with the isotype (24 µg/mL mAb-5G2) and negative controls, the results of quantitative real-time PCR (qPCR) showed that the reduction of PCV2 DNA copies was dose-dependent of the mAb-1E7 (Fig. 1C). Moreover, the progeny PCV2 in the supernatants of PK-15 cells from 24 µg/mL mAb-1E7 treated group showed a nearly three-log_10_ reduction compared with those of the PCV2-inoculated PK-15 cells without mAb pretreatment (Fig. 1D). Meanwhile, the results of TCID_50_ also showed that the reduction of progeny PCV2 was dose-dependent of the mAb-1E7 (Fig. 1D). Collectively, these above data indicated that mAb-1E7 is a specific neutralizing antibody against PCV2b, which can effectively inhibit PCV2b infection in PK-15 cells.

Third, using the BABL/c as a PCV2b infection animal model (34–36), the neutralization of mAb-1E7 was also evaluated *in vivo*. After the PCV2b (TCID_50_ = 10^6^, 100 µL) was incubated with mAb-1E7 (24 µg, 50 µL), isotype control mAb-5G2, or PBS at 37°C for 1 h, the mixtures were separately injected intramuscularly into the mice. Serum and lymph node samples were collected 4 weeks after immunization, as seroconversion against PCV2 is expected to take 2–4 weeks (37). At 4 weeks post-inoculation (wpi), all mice seroconverted to positive for anti-PCV2 antibody in the PBS/PCV2 and mAb-5G2/PCV2 groups (Fig. 1E). In contrast, the mice in the mAb-1E7/PCV2 group exhibited no detectable seroconversion, which was same as no virus challenged group (Fig. 1E). Moreover, the results of qPCR showed that the viral DNA copies in the serum and lymph nodes samples from the mAb-1E7/PCV2 group were significantly lower than the ones from the PBS/PCV2 and mAb-5G2/PCV2 groups (Fig. 1F and G). These results indicated that the mAb-1E7 could block the mice from PCV2b infection.

Based on the above data, the mAb-1E7 is a neutralization antibody against PCV2, indicating that it recognized a neutralizing epitope of PCV2-Cap. And then, the mAb-1E7 was used to produce an anti-idiotype nanobody.

### Construction of the VHH library and screening of nanobodies against mAb-1E7

**Fig 2:**
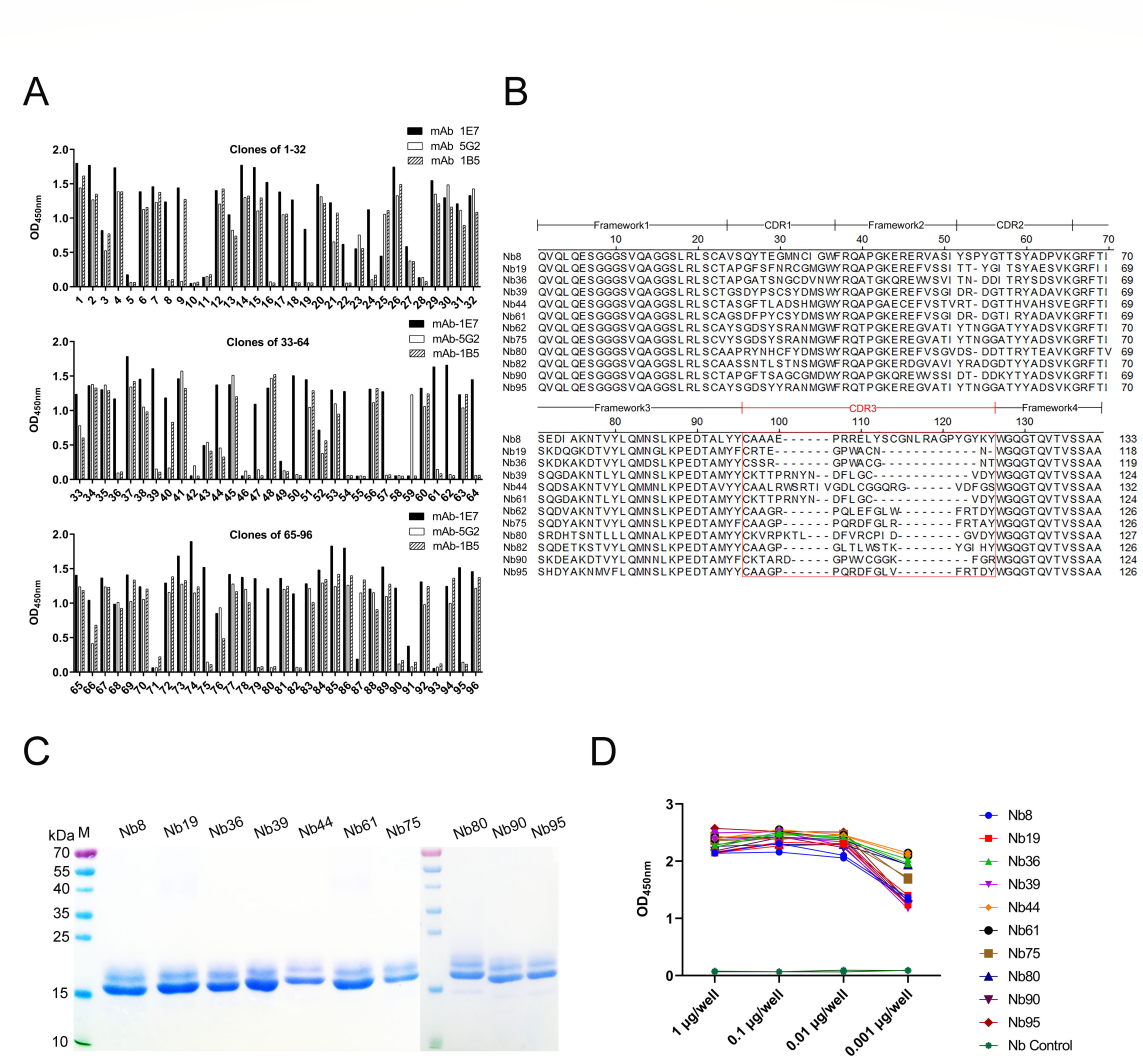
Screening of nanobodies against mAb-1E7 from the VHH library and producing nanobodies against mAb-1E7 by Pichia pastoris expression system. (**A**) Identification of the periplasmic extracts from the 96 clones specifically binding to the mAb-1E7 with an indirect ELISA. A total of 23 clones were positive. (**B**) Alignment of the amino acid sequences of 12 nanobodies against mAb-1E7. (**C**) SDS-PAGE analysis of the 10 specific nanobodies expressed with the Pichia pastoris system and purified by Ni-Resin. (**D**) Titers of the 10 purified nanobodies reacting with the mAb-1E7 by indirect ELISA.

After the camel was immunized with mAb-1E7 five times, the results of indirect ELISA showed that the titer of the anti-mAb-1E7 antibody in the serum samples could reach up to 1:10^6^ (Fig. S2A). After the total RNA was extracted from the peripheral blood lymphocytes (PBLs) and reverse transcribed into cDNA, the VHH gene with an expected size of 400 bp was successfully amplified by nested PCR. A phage display VHH library against mAb-1E7 was constructed, which contained approximately 7 × 10^9^ individual transformants. Then, the insertion rate of the VHH gene was determined to be 96% by PCR in 48 clones (Fig. S2B). Subsequently, the sequences of 48 clones showed good diversity in this library (data not shown). After three rounds of panning, the phages exhibiting mAb-1E7-specific VHHs were enriched, and the positive/negative clones (P/N) ratio increased from 4 to 7.5 × 10^2^ (Table S1). Then, 96 individual colonies from the third round plates were selected. Their peripheral crude extracts were produced and tested with an indirect ELISA using mAb-1E7 as a coated antigen. Meanwhile, mAb-5G2 and mAb-1B5, as the indirect ELISA coating antigen, were used as the isotype antibody control (38, 39). The results showed that a total of 23 positive colony extracts could specifically bind to mAb-1E7 (Fig. 2A). Then, those positive clones were sequenced, and based on the amino acid sequence alignments from the 23 clones, 12 nanobodies were screened and were separately named Nb8, Nb19, Nb36, Nb39, Nb44, Nb61, Nb62, Nb75, Nb80, Nb82, Nb90, and Nb95 (Fig. 2B).

### Production of nanobodies against mAb-1E7 with Pichia pastoris expression system

To produce the 12 nanobodies against mAb-1E7, they were all expressed with the Pichia pastoris expression system. Using mAb-1E7 as the primary antibody, Western blot analysis showed that all 12 nanobodies were secreted into the culture medium and still bound to the mAb-1E7 (Fig. S2C). Additionally, the specific bindings of 12 nanobodies to mAb-1E7 were also determined with the indirect ELISA. The results showed that 10 nanobodies could react with mAb-1E7 but not with isotype control mAb-5G2 (Fig. S2D). The other two nanobodies (Nb62 and Nb82) reacted with the two mAbs (Fig. S2D). The results showed that Nb62 and Nb82 have nonspecific recognition against isotype IgG control (Fig. S2D), which differs from what is shown in Fig. 2A. We think the reason may be that the secretion expression of nanobodies in the periplasmic extracts of *Escherichia coli* is low, but the secretion of nanobodies in the supernatant by yeast is high. Meanwhile, the results suggested that the 10 nanobodies may bind to variable regions of mAb-1E7, while Nb62 and Nb82 may bind to the constant region of mAb-1E7 and mAb-5G2. Then, the supernatants containing 10 nanobodies were purified with a Ni-Resin column according to the manual instructions. SDS-PAGE analysis showed that the 10 nanobodies were successfully purified with high purity (>98%) (Fig. 2C). Then, using the different amounts of 10 purified nanobodies as coated antigens, the indirect ELISA results showed that the 10 nanobodies still specifically bound to mAb-1E7 (Fig. 2D).

### Nb61 as an anti-idiotype nanobody mimicking PCV2-Cap

**Fig 3:**
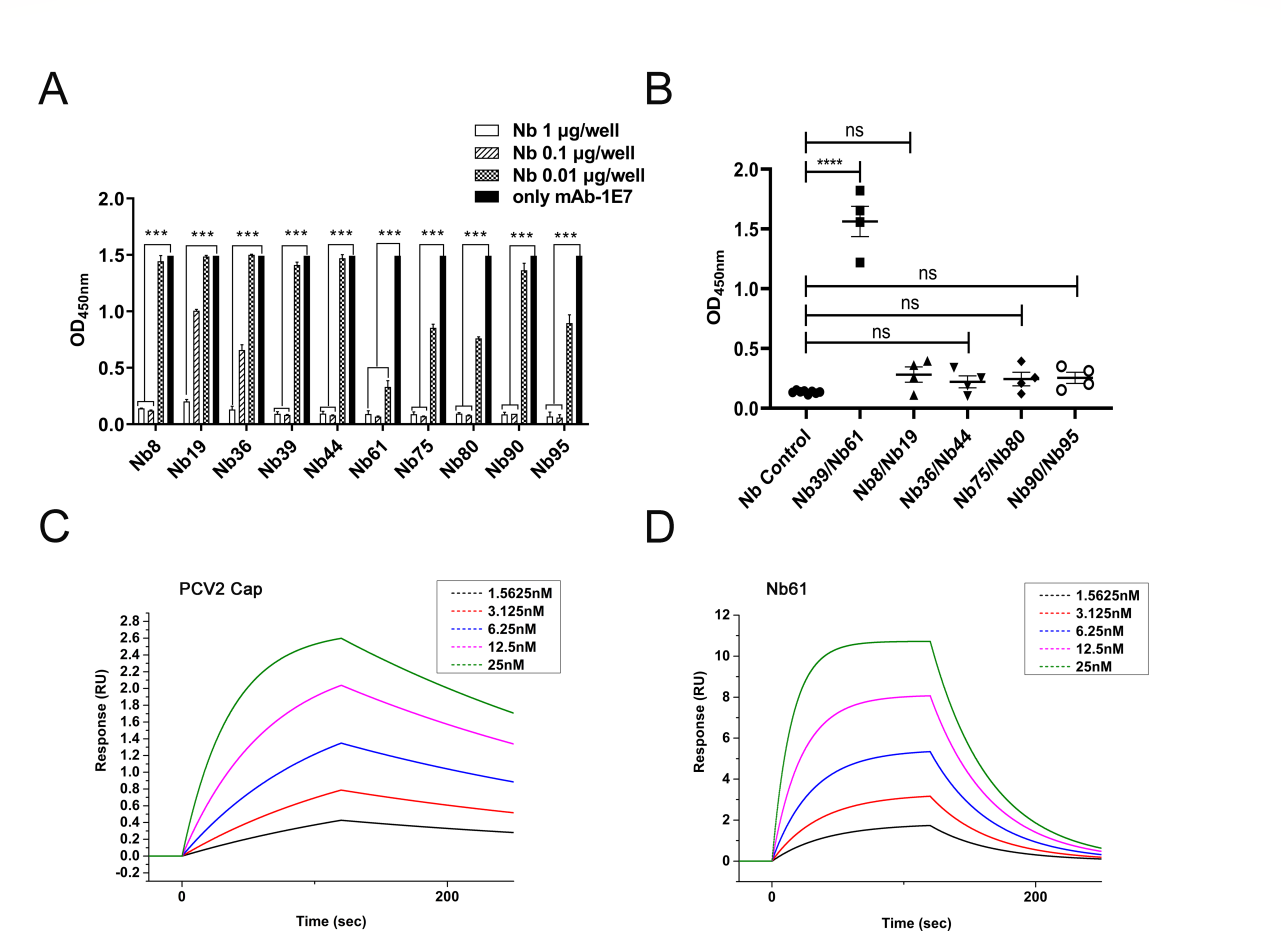
Nb61 as anti-idiotype nanobody mimicking PCV2-Cap. (**A**) Nanobodies block mAb-1E7 binding to PCV- Cap protein (coating with 400 ng/well) by blocking ELISA. (**B**) Detection of antibodies against PCV2-Cap (Ab3) in the mice immunized with the nanobodies by indirect ELISA. Each symbol represents one mouse, and the horizontal lines indicate the geometric mean of each group. The affinities of mAb-1E7 bind separately to PCV2-Cap (**C**) and Nb61 (**D**). Surface plasmon resonance (SPR) sensorgrams of monomeric PCV2-Cap and Nb61 binding to and subsequent dissociation from immobilized mAb-1E7 at five doses (1.5625–25 nM).

The blocking ELISA was first designed and performed to screen the nanobodies mimicking the PCV2-Cap. Using the nanobodies as the protein to block the mAb-1E7 binding to PCV2-Cap, the results showed that all 10 nanobodies could block the reaction between mAb-1E7 and PCV2-Cap (Fig. 3A; Fig. S3), suggesting that the binding sites between mAb-1E7 and nanobodies were within or close to the binding site of mAb-1E7 with PCV2-Cap.

To further determine the nanobodies as Ab2β, which can be used as a surrogate antigen to elicit Ab3, the 10 nanobodies were paired randomly into five groups (Table S2) and as antigens to immunize the mice for producing Ab3 (reacting with PCV2-Cap). The mice sera were collected after the last immunization. Then, Ab3 in sera was determined with an indirect ELISA using the recombinant PCV2-Cap (400 ng/well) as a coating antigen. The results showed that Ab3 binding to PCV2-Cap could be detected in the sera of BALB/c mice immunized with Nb61 and Nb39 (Fig. 3B). However, the Ab3 was not detected in the sera of BALB/c mice immunized with the other nanobodies (Fig. 3B). Therefore, the nanobodies Nb61 and Nb39 were initially identified as the Ab2β that mimics the PCV2-Cap. The amino acid sequences of Nb39 and Nb61 showed high identity, and only 3 aa were different at aa 23, 27, and 57. The blocking effects of Nb61 and Nb39 were compared, and Nb61 showed a stronger blocking effect (Fig. 3A; Fig. S3). So, Nb61 was selected for further experiments.

Subsequently, Surface Plasmon Resonance (SPR) was used to determine the binding affinity of mAb-1E7 to PCV2-Cap and Nb61. The results showed that the binding kinetics of mAb-1E7 with the two antigens were similar (Fig. 3C and D). The average association rate constants (*K*_a_) for Nb61 and PCV2-Cap were separately 18.6 and 9.84 × 10^5^ M^−1^ s^−1^, and their respective dissociation rate constants (*K*_d_) were 2.22 × 10^−2^ s^−1^ and 3.25 × 10^−3^ s^−1^. Thus, equilibrium dissociation constants (KD) separated 12 nM for Nb61 and 3.3 nM for PCV2-Cap. These measurements showed that mAb-1E7 binding to Nb61 was as strong as PCV2-Cap.

### Immunization with Nb61 protects against PCV2 infection *in vivo*

**Fig 4:**
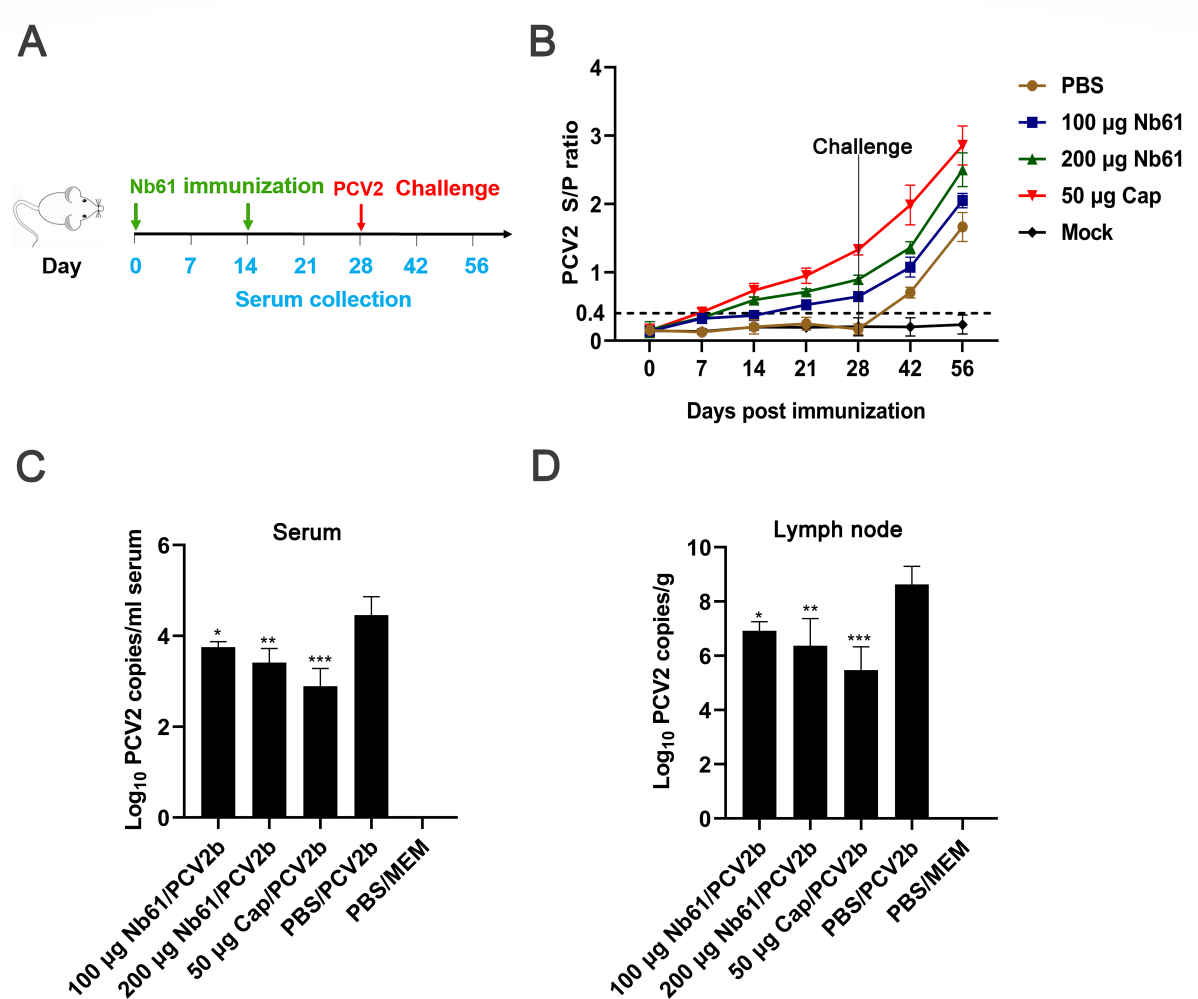
Immunization with Nb61 protects mice against PCV2 infection. (**A**) Schematic illustration of the animal experiment protocol. (**B**) PCV2 antibodies (Ab3) were detected against PCV2-Cap in the mice after immunization with Nb61 and infection with PCV2b. Detection of PCV2 DNA copies in sera (**C**) and lymph node (**D**) from the mice inoculated with PCV2b.

As shown above, Ab3 raised by immunization with Nb61 has a similar antigenic binding ability to mAb-1E7, indicating that the Nb61 can mimic an epitope of PCV2-Cap. Therefore, the Nb61 as the immunogen to protect the animals against PCV2 infection was analyzed. Two kinds of animals, including BABL/c mice and pigs, were used to perform the experiments *in vivo*. First, the immunization procedures were designed using BABL/c mice as PCV2b infection animal models (Fig. 4A; Table S3). The Ab3 in the immunized mice was determined by PCV2-dCap-ELISA Kit (JNT, Beijing, China), and the cutoff value of the S/P ratio was 0.4. At 0 and 7 days post-immunization (dpi), all mice were negative for PCV2-specific antibodies. In groups immunized with Nb61 or PCV2-Cap, all mice were seropositive at 14 dpi. In the group immunized with 200 µg Nb61, the mice had a higher S/P ratio during 14–56 dpi than those in the group with 100 µg. However, all the mice from the two groups were lower than the ones of the group immunized with the amount of 50 µg PCV2-Cap (positive control group) (Fig. 4B). These above results indicated that the Nb61 could elicit the Ab3 reacting with PCV2-Cap and the titers were lower than the Ab1 produced by the PCV2-Cap. To further evaluate whether the Ab3 can protect mice against PCV2b infection, viruses in the sera and lymph nodes of the infected mice were determined. At 28 days after the PCV2b challenge, the results of qPCR showed that the copies of PCV2 DNA in the serum and lymph nodes from the mice in the 100 µg Nb61/PCV2b or 200 µg Nb61/PCV2b group mice were significantly lower than that of the PBS/PCV2b group but higher than the 50 µg PCV2 Cap/PCV2b group (Fig. 4C and D). Meanwhile, the PCV2 DNA copies of mice from the 200 µg Nb61/PCV2b group were lower than those of the 100 µg Nb61/PCV2b group (Fig. 4C and D). These results indicated that Nb61 can elicit antibodies against PCV2-Cap in mice and confer protection against PCV2b infection in a dose-dependent manner.

**Fig 5:**
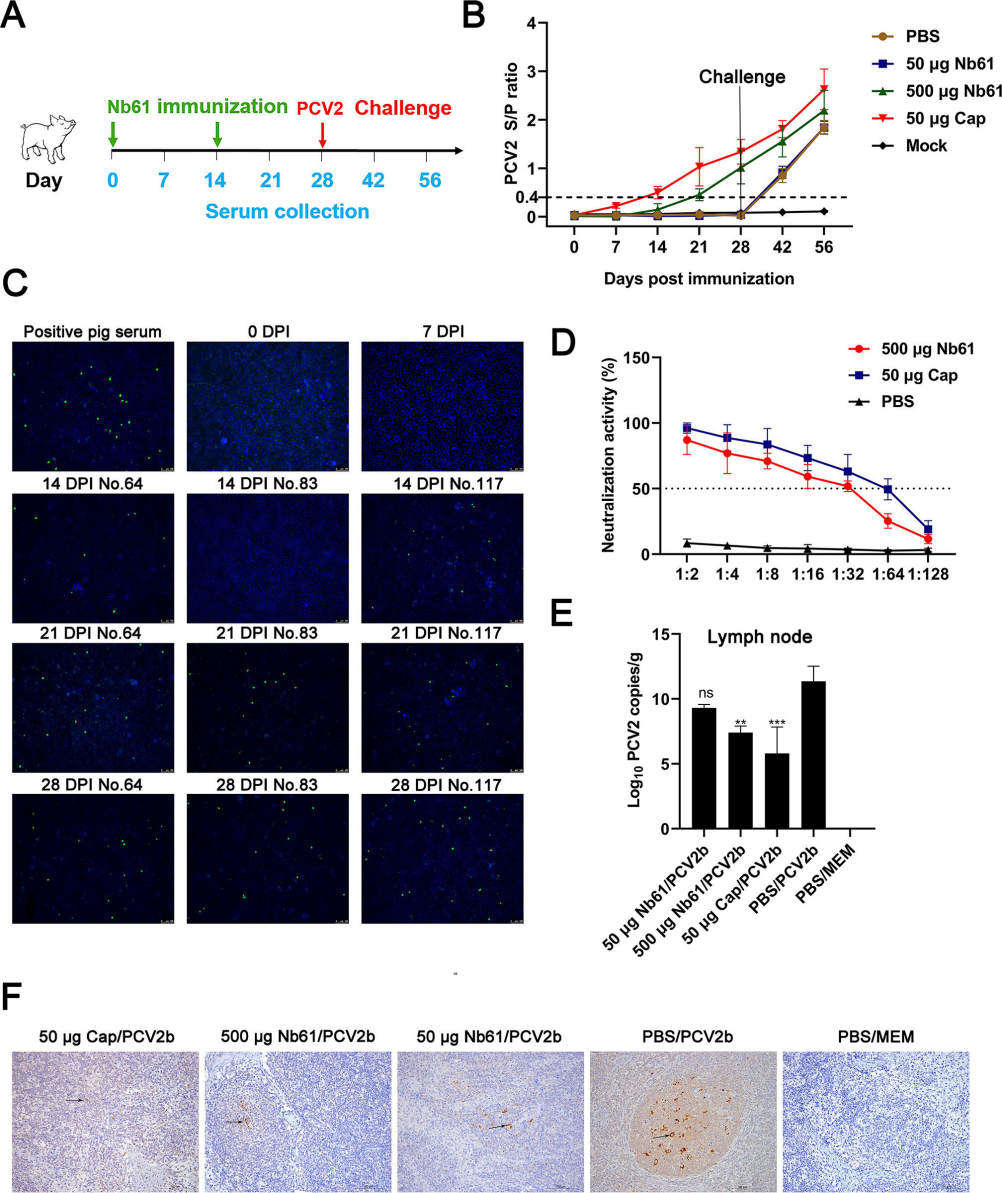
Immunization with Nb61 protects pigs against PCV2 infection. (**A**) Schematic illustration of the animal experiment protocol. (**B**) PCV2 antibodies (Ab3) were detected against PCV2-Cap in the pigs after immunization with Nb61 and infection with PCV2b. (**C**) Detection of serum samples from pigs immunized with 500 µg Nb61 binding to PCV2-infected PK-15 cells by IFA. (**D**) Neutralization activity of sera from the pigs immunized with Nb61 and PCV2-Cap. (**E**) Detection of PCV2 DNA copies in the lymph nodes of pigs after infection with PCV2b. (**F**) Detection of PCV2 in lymph node tissues by immunohistochemical staining.

Second, the pigs were used to verify that Nb61 triggers an effective Ab3 antibody response *in vivo* and protects against PCV2b infection. The pigs were divided into five groups (*n* = 3, Table S4), and immunization procedures were designed (Fig. 5A). Ab3 was also determined with a PCV2-dCap-ELISA Kit. At 0 and 7 dpi, all pigs were negative for PCV2-specific antibodies. Pigs in the group immunized with 500 µg Nb61 were seropositive on day 21 after the primary immunization, while pigs immunized with 50 µg PCV2-Cap were seropositive at 14 dpi. Pigs in the 50 µg-PCV2-Cap group had a higher S/P ratio during 14–56 dpi than the 500 µg-Nb61 group. Pigs in the 50 µg-Nb61 group and PBS group were negative for anti-PCV2-Cap antibodies during 0–28 dpi (Fig. 5B). To test whether serum samples from pigs of the 500 µg-Nb61 group reacted with PCV2-infected PK-15 cells by IFA, the sera from three immunized pigs (No. 64/83/117) were collected at 0 (before immunization), 7, 14, 21, and 28 dpi. The results showed that the serum samples at 0 and 7 dpi did not bind to PCV2b-infected PK-15 cells (Fig. 5C). The sera from No. 64 and 117 pigs at 14 dpi could bind, and the sera from all three pigs at 21 and 28 dpi also bound (Fig. 5C). Then, the neutralization of the sera from three pigs at 28 dpi was determined in PCV2b-infected PK-15 cells. The results showed that the pig sera from the 500 µg-Nb61 and 50 µg-Cap groups could effectively neutralize PCV2b infection. The neutralizing titer of pig sera from the 500 µg-Nb61 group was 2^5.059^, and the titer of pig sera from the 50 µg-PCV2-Cap groups was 2^6.35^ (Fig. 5D). The amounts of virus in the lymph node of infected pigs were assessed by qPCR. At 28 days after the PCV2b challenge, the PCV2 DNA copies in the lymph nodes of pigs from 500 µg-Nb61/PCV2b or 50 µg-PCV2-Cap/PCV2b group were significantly lower than that of the PBS/PCV2b group (Fig. 5E). IHC staining of PCV2 antigen was done on lymph node tissues of pigs at 56 dpi. The results showed that PCV2 antigens were detected in tissues from the 50 µg-Nb61/PCV2b and PBS/PCV2b group. PCV2 antigens were hardly detected in the 500 µg-Nb61/PCV2b and 50 µg-PCV2-Cap/PCV2b, and no PCV2 antigens were detected in PBS/MEM group (Fig. 5F). The number of PCV2 antigens in the lymph nodes of pigs from 500 µg-Nb61/PCV2b or 50 µg-PCV2-Cap/PCV2b group is significantly lower than that of the 50 µg-Nb61/PCV2b and PBS/PCV2b group. These results indicated that Nb61 can elicit antibody responses in pigs and confers protection against PCV2b infection.

### Nb61 mimics the ^75^NINDFL^80^ epitope of PCV2 Cap

**Fig 6:**
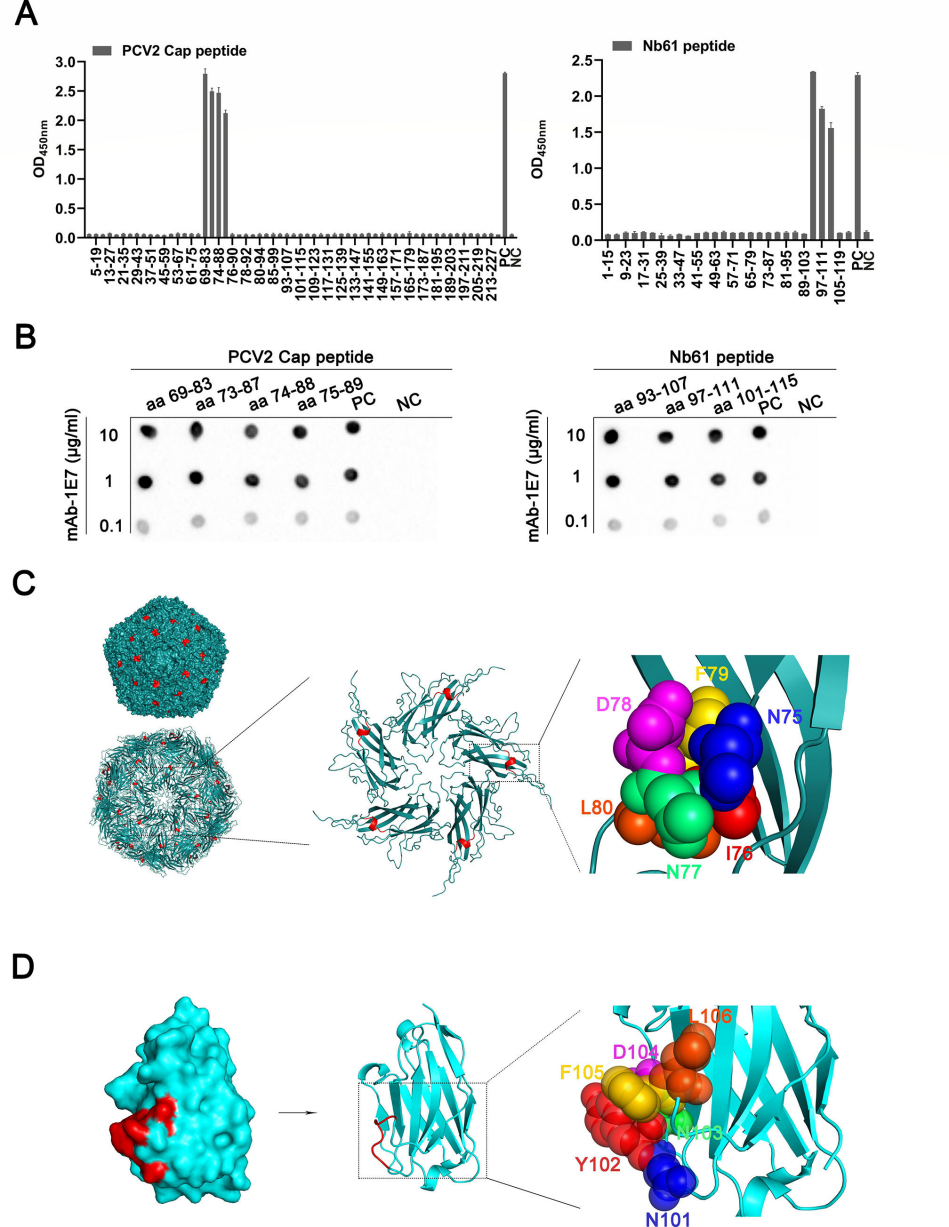
Mapping epitopes recognized by mAb-1E7 in the PCV2-Cap and Nb6. (**A**) Detection of different peptides from PCV2-Cap and Nb61 reacting with mAb-1E7 by indirect ELISA. PC, PCV2 Cap/Nb61 was the positive control. NC, peptides of unrelated proteins, was the negative control. (**B**) Detection of different peptides from PCV2-Cap and Nb61 reacting with mAb-1E7 by dot immunoblotting. Different concentrations of mAb-1E7 interacted with the peptides of PCV2 Cap and Nb61. PC, PCV2 Cap/Nb61 was the positive control. NC, peptides of unrelated proteins, was the negative control. (**C**) The structure of PCV2 Cap forming virus-like particles is shown as surface and cartoon. The structure of the PCV2 Cap monomer is shown as a cartoon. The epitopes ^75^NINDFL^80^ are shown in red. The epitope ^75^NINDFL^80^ is shown as colored spheres after partially enlarging the PCV2 Cap monomer structure. (**D**) The structure of Nb61 is shown as surface and cartoon, and the epitopes ^101^NYNDFL^106^ are shown in red. The epitope ^101^NYNDFL^106^ is shown as colored spheres after partially enlarging the Nb61 structure.

The amino acid sequences of Nb61 and PCV2-Cap protein were compared using DNAman software. Alignments showed that the region (^101^NYNDFL^106^) on CDR3 of Nb61 is consistent with the five key amino acids (^75^NINDFL^80^) of PCV2-Cap (Fig. S4). So, we speculate that Nb61 mimics the ^75^NINDFL^80^ epitope of PCV2-Cap. To further determine that Nb61 is Ab2β mimicking the epitope of PCV2-Cap, the peptides scanning PCV2-Cap and Nb61 were separately synthesized. The 28 peptides with a 4 amino acid offset (Nb61) and 61 peptides with a 4 amino acid offset (PCV2-Cap) were synthesized, respectively (Tables S5 and S6). With these peptides as coating antigens and mAb-1E7 as the first antibody, the ELISA analysis showed that mAb-1E7 bound peptides of aa 69–83, aa 73–87, aa 74–88, and aa 75–89 of PCV2-Cap and peptides of aa 93–107, aa 97–111, and aa 101–115 of Nb61 (Fig. 6A). The dot immunoblotting results also showed that four peptides aa 69–83, aa 73–87, aa 74–88, and aa 75–89 of PCV2-Cap and three peptides aa 93–107, aa 97–111, and aa 101–115 of Nb61 could react with the mAb-1E7 (Fig. 6B). Based on these peptides scanning the amino acids, the results indicated that the ^75^NINDFLPPG^83^ region is the minimal length of the epitope in the PCV2-Cap and the ^101^NYNDFLG^107^ region is the minimal length of the Nb61. Above all, these results indicated that Nb61 mimics the ^75^NINDFL^80^ epitope of PCV2 Cap. Then, the AlphaFold2 server and PyMOL software were used to predict the spatial structure of the epitopes separately located in Nb61 and PCV2-Cap. The predicted results showed that the ^75^NINDFL^80^ of PCV2-Cap (Fig. 6C) and ^101^NYNDFL^106^ of Nb61 (Fig. 6D) were localized within surface-exposed loops of the structural domain. These findings strongly suggested that the structure of Nb61 enables effective exposure of critical epitopes, leading to the generation of antibodies with neutralization activity.

### Monoclonal antibody against ^101^NYNDFL^106^ of Nb61 neutralizing PCV2 infection

**Fig 7:**
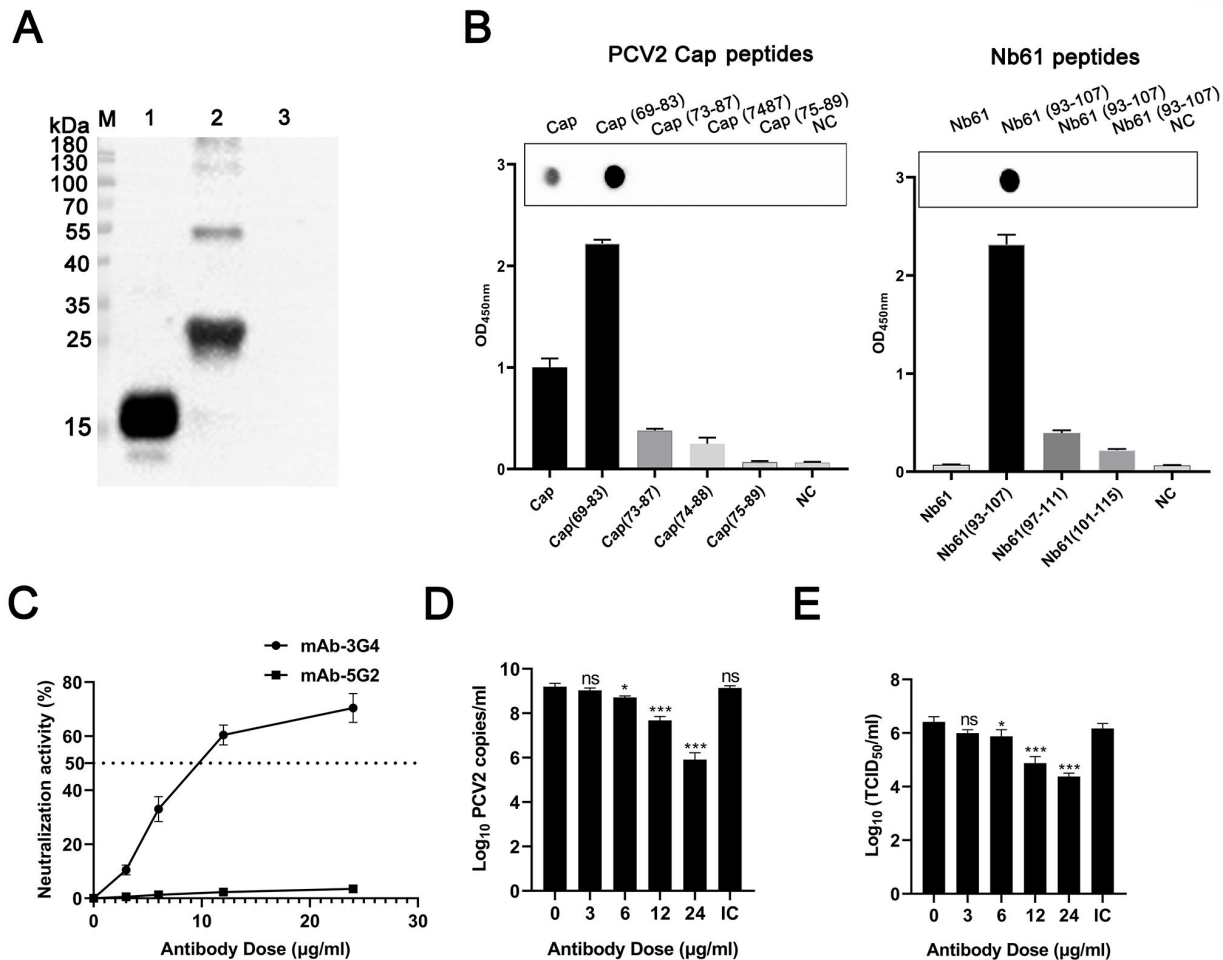
Neutralization analysis of mAb-3G4 against Nb61. (**A**) Detection of mAb-3G4 binding to PCV2-Cap and Nb61 by Western blot. M: Marker; lanes 1: Nb61; lanes 2: PCV2-Cap; lanes 3: sPRA protein as a negative control. (**B**) Detection of mAb-3G4 binding to different peptides from the PCV2-Cap and Nb61 by dot immunoblotting and indirect ELISA. BSA was the negative control (NC). (**C**) Neutralization activity of mAb-3G4 with different concentrations against PCV2b infection. PK-15 cells infected with PCV2b SH strain (100 TCID_50_) pretreated with purified mAb-3G4 at indicated concentrations. Mab-5G2 was as IgG1 isotype control (IC). The minimum antibody concentration that reduced the number of PCV2-infected cells by 50% was defined as the IC_50_ for the antibody. The dotted line indicates the cutoff value. Error bars indicate the standard deviations. (**D**) Detection of PCV2 DNA copies in infected PK-15 cells by qPCR. (**E**) Detection of viral titers in cell culture supernatant by TCID_50_.

Based on the above data, Nb61 can elicit antibodies against PCV2-Cap (called Ab3 based on Jernes' idiotype network theory) *in vivo* to confer protection against PCV2 infection. An extensive structural analysis was performed to identify the epitopes responsible for this observed mimicry to determine the idiotype network, including PCV2-Cap, mAb-1E7, Nb61, and Ab3. First, mAb against Nb61 was produced by traditional hybridoma technology. After immunizing four times with the Nb61, the titers of antibodies in the four mice were all over 1:10^7^ by detecting indirect ELISA (Fig. S5). A mAb against Nb61 was successfully screened and named mAb-3G4 by the hybridoma technique. Then, a Western blot was performed to verify the mAb-3G4 reacting with Nb61 and PCV2-Cap. The results showed that the mAb-3G4 could react with PCV2-Cap and Nb61 under denaturing conditions (Fig. 7A). Additionally, using the above-synthesized peptides scanning PCV2-Cap and Nb61 as coating antigen and mAb-3G4 as the first antibody, the results of dot immunoblotting and indirect ELISA showed that the mAb-3G4 recognizes the aa 93–107 (^93^YYCKTTPR**NYNDFL**G^107^) of Nb61 and the aa 69–83 (^69^VDMMRF**NINDFL**PPG^83^) of PCV2-Cap (Fig. 7B). Subsequently, the 50% inhibitory concentration (IC_50_) values of mAb-3G4 was also measured by virus neutralization assays. PCV2b strain SH (200 TCID_50_, 100 µL) was incubated with 100 µL of serially diluted mAb-3G4 for 1 h at 37°C. Then, the mixtures were separately added into PK-15 cells. At 48 hpi, the IFA results showed that when the concentration of mAb-3G4 increased to 10.41 µg/mL, the inhibitory concentration can reach up to 50% (Fig. 7C) similar to the mAb-1E7. To further confirm the neutralizing effect of mAb-3G4, the viral amount in the infected cells and progeny virus in cell culture supernatant were also determined. Compared with the isotype (24 µg/mL) and negative controls, the results of qPCR showed that the reduction of PCV2 DNA copies was dose-dependent of the mAb-3G4 doses (Fig. 7D). Moreover, the titers of progeny PCV2b in the supernatants from the 24 µg/mL mAb-3G4-treated group showed a nearly two-log_10_ reduction than those of the PCV2-inoculated PK-15 cells without mAb pretreatment. The results of TCID_50_ detection showed that the reduction of progeny PCV2 was also dose-dependent of mAb-3G4 (Fig. 7E). Collectively, these data indicated that mAb-3G4, like mAb-1E7, is a specific neutralizing antibody against PCV2b, which can effectively neutralize PCV2b infection in the PK-15 cells.

### Sequencing of antibody and molecular docking

**Fig 8:**
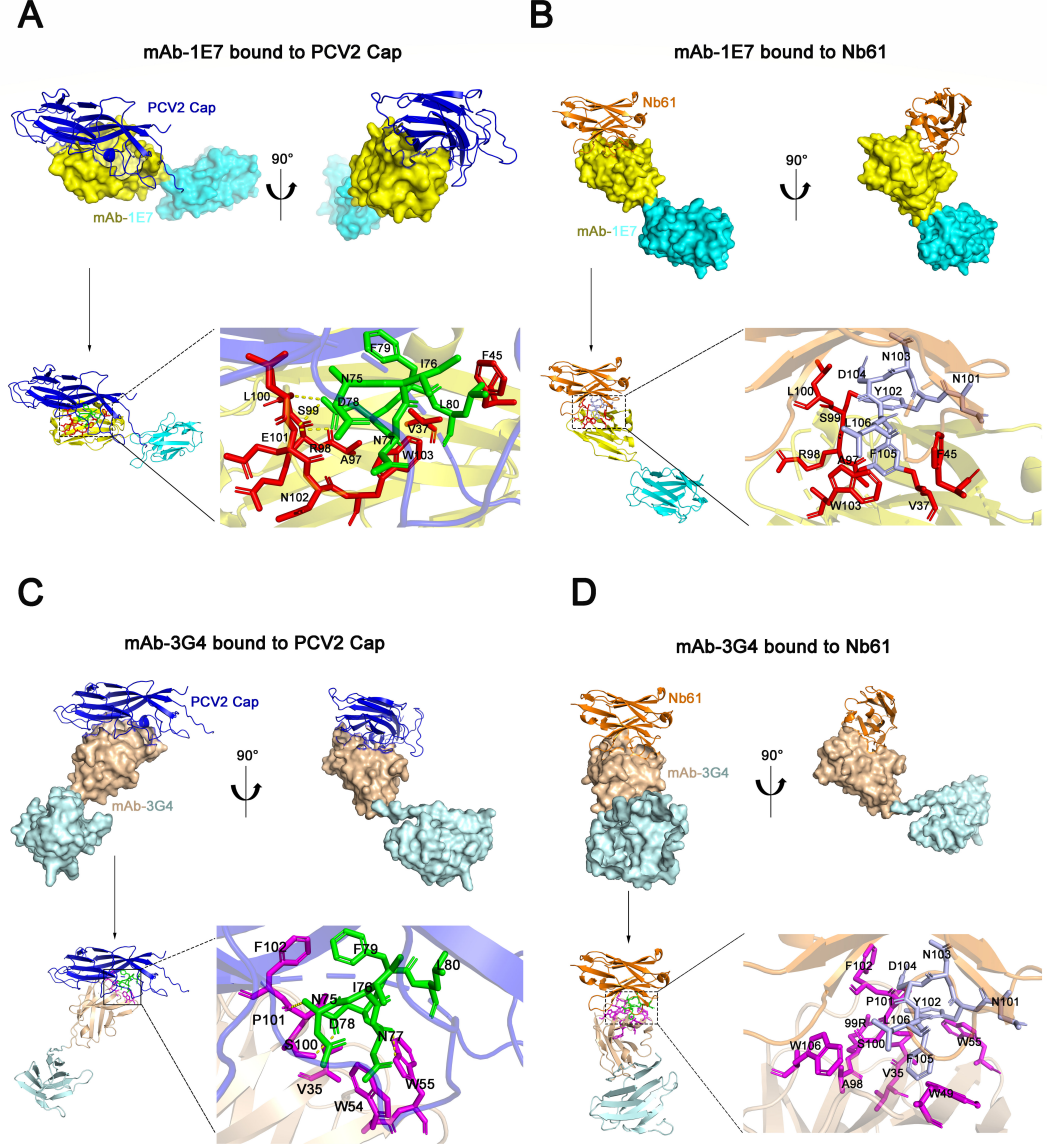
Structures analysis of mAb-1E7 and mAb-3G4 separately binding to PCV2-Cap and Nb61 by molecular docking. (**A**) Structure analysis of mAb-1E7 binding to PCV2-Cap (mAb-1E7 heavy chain: yellow, light chain: cyan, PCV2-Cap: blue). Stick representation of the interaction residues for mAb-1E7 (red) and PCV2-Cap (green). Yellow dashed lines denote the hydrogen bonds. (**B**) Structure analysis of mAb-1E7 binding to the Nb61 (Nb61: orange). Stick representation of the interaction residues from mAb-1E7 (red) and Nb61 (light blue). (**C**) Structure of mAb-3G4 binding to PCV2-Cap (mAb-3G4 heavy chain: wheat, light chain: pale cyan, PCV2-Cap: blue). Stick representation of the interaction residues from mAb-3G4 (magentas) and PCV2 Cap (green). Yellow dashed lines denote the hydrogen bonds. (**D**) Structure of mAb-3G4 binding to the Nb61 (Nb61: orange). Stick representation of the interaction residues for mAb-3G4 (magentas) and Nb61 (light blue).

The nucleotide and amino acid sequences of VH and VL of mAb-1E7 and mAb-3G4 were sequenced by the GenScript Biotech Corporation (Fig. S6). Then, the spatial structures of interactions among mAb-1E7, mAb-3G4, PCV2-Cap, and Nb61 were predicted based on their sequences and binding sites determined by the molecular docking software. The ZDOCK algorithm generated matches between the models based on their surface complementarity (40, 41). The amino-acid residues’ contact interface was identified as the one in which any atom of the residue is separated from any binding partner atom by a distance **≤**5 Å. The results showed that the motifs V37, F45, A97, R98, S99, L100, E101, N102, and W103 located in the CDR3 of heavy chain of mAb-1E7 can form antigen-binding pocket and bind to the epitope ^75^NINDFL^80^ of PCV2-Cap (Fig. 8A). Among these amino acids, the motif N75 of PCV2-Cap directly forms hydrogen bonds with L100 and E101 of mAb-1E7 (Fig. 8A). The results of molecular docking between mAb-1E7 and Nb61 also showed V37, F45, A97, R98, S99, L100, and W103 of mAb-1E7 heavy chain bound to the ^101^NYNDFL^106^ of Nb61 (Fig. 8B). These results indicated that the motifs in the mAb-1E7 for binding to PCV2-Cap and Nb61 were similar. Subsequently, the spatial structures of mAb-3G4 binding to PCV2-Cap and Nb61 were also predicted by the docking software. The docking results showed that the motifs V35, H52, W54, W55, R99, S100, P101, F102, and F103 located in CDR3 of mAb-3G4 were involved in binding to ^75^NINDFL^80^ of PCV2-Cap (Fig. 8C). Within the epitope, the motifs N75, N77, and D78 of PCV2-Cap can form hydrogen bonds with P101, W55, and S100 of mAb-3G4 (Fig. 8C). The two docking results of mAb-1E7 and mAb-3G4 to PCV2-Cap also suggested that the spatial structures of the two mAbs binding to PCV2-Cap were similar (Fig. 8A and C). Similar structures were also observed in the docking of mAb-3G4 and mAb-1E7 binding to Nb61 (Fig. 8B and D). The motifs V35, W49, W55, A98, R99, S100, P101, F102, F103, and W106 located in the CDR3 of mAb-3G4 were involved in interaction with the ^101^NYNDFL^106^ of Nb61 (Fig. 8D). Overall, these data demonstrated that the Nb61 mimicked the neutralizing epitope ^75^NINDFL^80^ of PCV2-Cap in amino acids, structure and function, which in turn thus allows mAb-3G4 (Ab3) to structurally and functionally mimic mAb-1E7 (Ab1).

## DISCUSSION

Since the first vaccine was developed more than 200 years ago, vaccination has greatly reduced the global burden of infectious diseases (42). However, because of the emergence of new and old pathogens, more novel technologies still need to be developed for quickly designing and producing vaccines. Jernes’ idiotype network theory provides a strategy for developing an anti-idiotype vaccine, which can solve the problem that some pathogens are difficult and dangerous to culture *in vitro* (6, 43). Anti-idiotype vaccines would eliminate the complications of using killed, attenuated, or subunit fractions of organisms as vaccines. Some previous studies documented that anti-idiotype antibodies can be used as an antigen to trigger the immune response against infectious pathogens and tumor-associated antigens (44–46). However, the traditional methodology for developing anti-idiotype vaccines has several drawbacks: (i) the production of ant-idiotype mAbs is challenging in syngeneic systems; (ii) making anti-idiotype antibodies that mimic small ligands is challenging; and (iii) it is difficult and expensive to produce the anti-idiotype antibody with expression system *in vitro* for following commercialization. Therefore, until now, there has been no commercial anti-idiotype vaccine. The researchers have been searching for the appropriate strategies to overcome most of the above limitations for developing anti-idiotype vaccines (43, 47). In the present study, we designed a novel, simple, straightforward strategy to generate molecular mimicry, taking advantage of nanobodies. Using the PCV2 as a viral model, the anti-idiotype nanobody mimicking PCV2-Cap was successfully produced and can elicit the neutralization antibodies in the pigs and mice to defend against viral infection. The results indicated that nanobodies are a good replacement for traditional monoclonal antibodies for developing anti-idiotype vaccines. Based on the advantages of nanobodies, including small molecules, low cost of production *in vitro,* and easy screening, the anti-idiotype vaccine using nanobody as a reagent will have a very good application in the future.

Previously, anti-idiotypic nanobodies have been employed to substitute toxins in immunoassays to improve operator and environmental safety, assay uniformity, and cost reduction (48, 49). Alvarez-Rueda et al. showed that nanobody targeting trastuzumab can induce anti-HER2 antibodies in mice, inhibiting cell viability (50). However, there were few reports about anti-idiotypic nanobodies mimicking antigens to induce immune responses. No studies showed that the anti-idiotypic nanobodies can induce an effective immune response *in vivo*. In the present study, the Nb61 was screened and was an anti-idiotype nanobody mimicking PCV2-Cap. The Nb61 was against the hypervariable region of neutralization mAb-1E7, and then, it mimics the neutralizing epitope ^75^NINDFL^80^ of PCV2-Cap. Importantly, the Nb61 can induce neutralizing antibodies and protect the pigs and mice against PCV2 infection. Then, our research about anti-idiotype nanobody Nb61 provided a novel strategy for developing vaccines against PCV2 infection in pig flocks.

Broadly neutralizing antibodies (bNAbs) are powerful tools for treating viral infections in humans and animals. They are especially useful for the influenza virus and coronavirus, which were easily genetic variations (51). Broad-spectrum vaccines are the most promising vaccines for the viruses. Theoretically, the epitopes recognized by the bNAbs can provide a basis for designing broad vaccines. However, preparing immunogens based on the conserved epitopes is very challenging, which are usually conformational and contain two or more exposed loops. So, it takes work to produce antigens to represent these epitopes. Additionally, even if the epitope is linear, the peptide corresponding to the sequences of epitopes usually cannot cause an effective immune response. Previous studies documented that anti-idiotypic antibodies can mimic the epitopes and induce neutralizing responses against virus infection (52). Anti-idiotype vaccines may be an ideal strategy for developing broad-spectrum vaccines. In the present study, we used the neutralizing antibody mAb-1E7 against PCV2 as a template, and then, Nb61 of the Ab2β anti-idiotype nanobody was obtained. Subsequently, we determined that the epitope ^75^NINDFL^80^ recognized by the mAb-1E7 was highly conserved among the different strains of PCV2a, PCV2b, PCV2d, PCV2c, and PCV2e (Fig. S7). The amino acids of epitopes are the same as the ^101^NYNDFL^106^ of Nb61, and we speculated that the Nb61 could also induce immune responses against different isolates of PCV2.

In this study, Nb61 mimics the neutralizing epitope ^75^NINDFL^80^ of PCV2-Cap and induces neutralizing antibodies to protect the pigs against PCV2 infection. More than 40 years ago, Jerne et al. proposed the “network theory,” in which anti-idiotypic antibodies could structurally resemble an antigen and mimic immunogenic activities (7, 53). In recent years, antigen mimicry has been defined at the molecular level for some antigens. Using hen egg lysozyme (HEL) as a model antigen, structural immunologists discovered that both the anti-HEL antibody (D1.3) and its anti-idiotypic antibody (E5.2) could bind to the D1.3 combining site (54). A previous study also documented that an anti-idiotype Fab E1 can partially mimic the E protein of Dengue virus (55). Few reports have confirmed that anti-idiotype antibodies completely mimic the antigen in structure and function (56). In the present study, to document that Nb61 truly mimics an epitope of PCV2 Cap, the interactions among mAb-1E7, mAb-3G4, and PCV2-Cap, Nb61 were further characterized by peptides synthesis and molecular docking. Interestingly, the CDR3 of Nb61 mimics the shape and character of the neutralizing epitope ^75^NINDFL^80^ of PCV2-Cap, and they have similar sequences. Meanwhile, the docking results showed that the amino acid residues of mAb-1E7 (Ab1) and mAb-3G4 (Ab3) separately binding to PCV2-Cap and Nb61 were also similar. These results suggested that the mAb-3G4 (Ab3) structurally and functionally mimics mAb-1E7 (Ab1). Because the mAb-3G4 was produced using the Nb61 and mAb-1E7 used PCV2-Cap, the Nb61 mimics PCV2 Cap in functional conformation and amino acid sequence level. As we know, it is the first time to identify that anti-idiotype nanobody functionally and structurally mimics the antigen.

Meanwhile, the docking results also showed that the amino acid residues of mAb-1E7 (Ab1) and mAb-3G4 (Ab3) separately binding to PCV2-Cap were similar but not identical. Furthermore, the affinity of the two monoclonal antibodies to PCV2-Cap cannot be identical. Therefore, the screened mAb-3G4 (Ab3) has neutralization activity that is not as optimal as mAb-1E7 (Ab1). The ^101^NYNDFL^106^ epitope of Nb61 mimics the ^75^NINDFL^80^ epitope of PCV2-Cap. Among the amino acids of the epitope, one amino acid is different and may be the reason that the neutralization activity of the mAb-3G4 (Ab3) is not as optimal as the one of mAb-1E7 (Ab1). In the future, the tyrosine in the Nb61 (Ab2) epitope (^101^NYNDFL^106^) will be mutated to isoleucine in the PCV2-Cap, and then, it was used to screen Ab3. The screened Ab3 may have the same neutralization activity as mAb-1E7 (Ab1).

Vaccination is now a major tool for controlling PCV2 infection in the pig farm. Five commercial vaccines against PCV2 exist, including two inactivated and three subunit vaccines (30). The PCV2 stocks in inactivated vaccines are usually produced in PK-15 cells, but viral titers in PK-15 cells are very low (57). Most of the subunit vaccines consisted of PCV2-Cap, which was expressed in the baculovirus system. However, the PCV2-Cap expressed with a baculovirus system is laborious and expensive (58). Clinical implementation of multiple immunization strategies has been effective in controlling the large-scale development of the disease, but regional morbidity and mortality continue to be reported. So, a new, effective, low-cost strategy for vaccine production of PCV2 is necessary. The present study successfully produced the anti-idiotype nanobody (Nb61) mimicking PCV2-Cap. We demonstrated that Nb61 was an effective immunogen against PCV2b infection in mice and pigs. Both mice and pigs immunized with Nb61 can stimulate the seroconversion of immune individuals. After challenge, the viral load in the serum samples and lymph nodes of mice and pigs decreased significantly. Meanwhile, PCV2 antigen in lymph nodes of pigs decreased significantly. The neutralization titer of serum antibody of pigs immunized with Nb61 was 2^5.059^. These results indicate that Nb61 elicits potent Ab3 *in vivo* to confer protection against PCV2b. Compared with traditional antibodies, nanobodies exhibit more attractive features for diagnostic applications, such as easy genetic manipulation and high stability. Therefore, the Pichia pastoris, an endotoxin-free host system for recombinant protein production, has been used to express Nb61 and achieved massive secretory expression, which laid the foundation for reducing vaccine costs. However, the same protection effects were achieved in mice and piglets immunized with low doses of PCV2-Cap and high doses of Nb61. The reason is that Nb61 only mimics one epitope of PCV2-Cap. In the future, to enhance the immunized effects of low doses of Nb61, it can be coupled or fused with lumazine synthase and ferritin to form the nanoparticles, which have been used to create multimeric vaccine molecules (59). Antigen display on the ferritin surface has many desirable features, such as the uniform presentation of 24 epitopes, as well as monodispersity, thermal, and pH stability of the ferritin nanocage (60). Representative ferritin-based vaccines target influenza, SARS-CoV-2, and Epstein-Barr viruses, and some have entered phase I clinical trials (61, 62). Lumazine synthase is a potential protein scaffold particle for the development of delivery and assembly vehicles (63). A recent study used the RBD from MERS using lumazine synthase nanoparticle delivery and found a neutralizing antibody response against MERS-CoV (64). During the 2019 coronavirus disease (COVID-19), it became obvious that nanoparticle delivery might be used to administer safe and efficacious vaccinations (65, 66).

**Fig 9:**
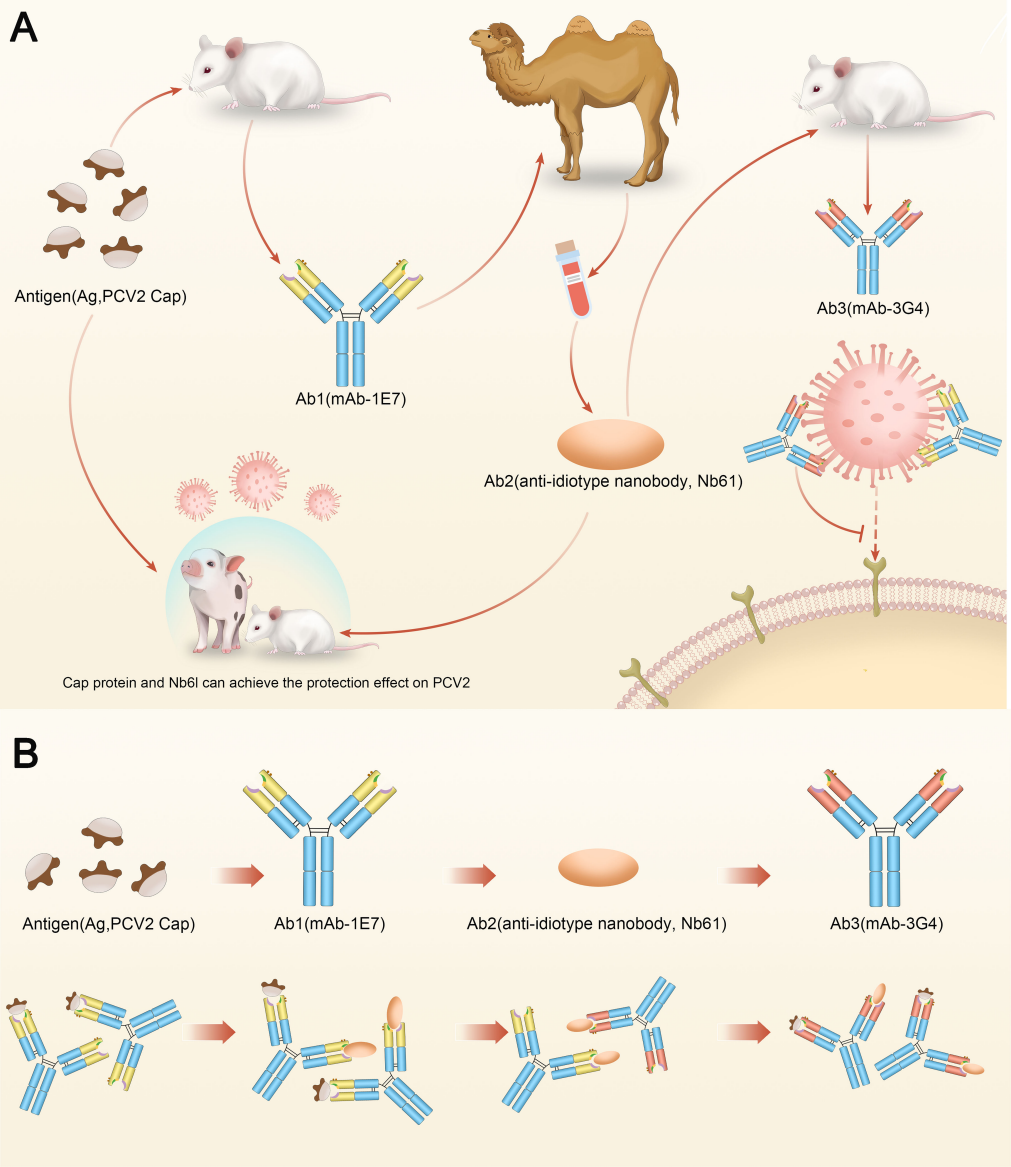
Schematic diagram. (**A**) Anti-idiotype nanobody Nb61 functionally mimics PCV2-Cap. Anti-idiotype nanobody Nb61 can induce neutralizing antibodies and protect the mice and pigs against PCV2 infection. (**B**) Idiotypic network theory: mAb-1E7 (Ab1) against PCV2-Cap (Antigen), Nb61 (Ab2) against mAb-1E7, and mAb-3G4 (Ab3) against Nb61. Nb61 mimicking PCV2-Cap can bind to mAb-1E7. mAb-3G4 mimicking mAb-1E7 can bind to PCV2-Cap.

In summary, an anti-idiotypic nanobody (Nb61) mimicking the PCV2-Cap was screened and can provide protection immune responses against PCV2 infection in the pigs and mice, which is the same as the PCV2-Cap. Mechanically, Nb61 mimics a neutralizing epitope ^75^NINDFL^80^ in PCV2-Cap and elicits potent Ab3 *in vivo* to confer protection against PCV2 infection (Fig. 9). We further confirmed that the anti-idiotype vaccines can be developed based on the “Jernes’ idiotype network theory” using nanobody technology. More importantly, using the anti-idiotype nanobody, vaccines against especially dangerous pathogens can be easily and effectively produced using the neutralization mAb.

## MATERIALS AND METHODS

### Cells and viruses

PK‐15 cells (ATCC CCL-33) were grown in Minimum Essential Medium (MEM) (Gibco, Grand Island, NY, USA) containing 10% fetal bovine serum (FBS) (Gibco, Grand Island, NY, USA), 0.1 mg/mL streptomycin and penicillin. SP2/0 cells were cultured in RPMI 1640 (Gibco, Grand Island, NY, USA) containing 10% FBS, 0.1 mg/mL streptomycin, and penicillin and were used for mAb preparation. PCV2b strains SH (GenBank accession number: HM038027) were propagated in the PK‐15 cells. The study used two mAbs, 5G2 (39) and 1B5 (38), as an isotype control. The mAb-1E7 was a gift from Pro. Cai of Harbin Veterinary Research Institute, Chinese Academy of Agricultural Sciences.

### Construction of VHH library and screening of nanobodies

A healthy 4-year-old Bactrian camel was immunized by subcutaneous route with the purified mAb-1E7 based on the previous procedures (67, 68). Briefly, 2 mg mAb-1E7 (1 mg/mL) was mixed with an equal volume of Freund’s complete adjuvant for the first immunization and the same volume of Freund’s incomplete adjuvant for the following four immunizations. The titration of antibodies against mAb-1E7 in the serum samples from the last immunization was tested with an indirect ELISA. The ELISA plates were coated with the purified mAb-1E7 and incubated overnight at 4°C. After being washed three times with PBS'T (0.5% Tween-20 in PBS), the plates were blocked with the blocking buffer (2.5% skimmed-milk in PBS'T). Then, the different dilutions (1:10^3^, 1:10^4^, 1:10^5^, and 1:10^6^) of sera were added to the plates, followed by rabbit anti-camel antibody and Peroxidase-AffiniPure goat anti-rabbit IgG antibody. The reaction was colored with tetramethylbenzidine (TMB). Finally, 3 M H_2_SO_4_ was added to stop the colorimetric reaction, and the OD_450nm_ values were read using an automated ELISA plate reader (BioTek Instruments, Inc.).

After the last immunization, the PBLs were extracted from 250 mL blood samples of immunized camel by Leucosep tubes (Greiner Bio-One, Germany) for library construction. Total mRNA was extracted from 5 × 10^7^ PBLs and used for cDNA synthesis by reverse transcriptase with the Olig (dT)_18_ primers. The VHH genes were amplified with the nested PCR using two primer pairs, CALL001, CALL002, and VHH-FOR, VHH-REV, according to a previous description (Table S7) (68, 69). Then, the nested PCR products were ligated into phagemid vector pMECS, which were digested with *Pst*I and *Not*I endonucleases. The recombinant phagemids were electro-transformed into freshly competent *E. coli* TG1 cells, and the positive rate of the library was determined by PCR amplification with primers p5E-FOR and VHH-REV (Table S7) (68). Finally, 48 clones were randomly selected for sequencing to analyze the library’s diversity.

To screen nanobodies against the mAb-1E7, three rounds of screening and phage rescuing were performed with an indirect ELISA according to the previous description (68). The purified mAb-1E7 was used as the coating antigen in the indirect ELISA for bio-panning. After three rounds of screening, the specific phage particles against mAb-1E7 were enriched and evaluated with polyclonal phage ELISA. Then, 96 clones were randomly selected from the third round plates and grown in liquid culture. Then, their periplasmic extracts were tested by the indirect ELISA to detect specific nanobodies against mAb-1E7. Finally, all positive clones were sequenced and classified based on their amino acid sequence.

### Expression and identification of different nanobodies against mAb-1E7

After the nanobodies against mAb-1E7 were screened, they were all expressed with the Pichia pastoris system (Invitrogen, USA). First, the genes encoding nanobodies were amplified using the primers pPICZαA-Nbs-F and pPICZαA-Nbs-R (Table S7) and ligated into the vector pPICZαA (Invitrogen, USA). After the recombinant vectors were digested by *Sac*I (TaKaRa, China), the linearized pPICZαA-Nbs were electro-transformed into the X-33 Pichia pastoris strain and placed in YPD plates with 100 µg/mL Zeocin (Gibco, USA). Then, the positive X-33 colonies containing the plasmids were identified by PCR. After the recombinant strains were activated in YPD medium for 2 days, their cultivation was enlarged in BMGY medium (containing 1% yeast extract, 2% peptone, 100 mM potassium phosphate, 1.34% YNB, 4 × 10^–5^% biotin, and 1% glycerol) for 1 day and then induced by 0.5% methanol for expression in BMMY (containing 1% yeast extract, 2% peptone, 100 mM potassium phosphate, 1.34% YNB, 4 × 10^–5^% biotin, and 0.5% methanol). Following 5 days of induction, the culture medium of Pichia pastoris was collected, and the pH was adjusted to 7.5 using 1 M Tris-Base for purification by Ni-Resin column (Roche, Mannheim, Germany). Then, the titers of purified nanobodies were determined by indirect ELISA. The ELISA plates were coated with the purified mAb-1E7, and different concentrations of nanobodies (1, 0.1, 0.01, and 0.001 µg/well) were added to the plates and followed to incubate with Rabbit anti-his tag antibody and Peroxidase-AffiniPure goat anti-rabbit IgG antibody. Finally, the OD_450nm_ values were read. The expression and purification of these nanobodies were analyzed by SDS-PAGE and Western blot.

### Blocking ELISA

To screen the nanobodies mimicking the PCV2-Cap, these nanobodies were used to block the binding of mAb-1E7 to PCV2-Cap by blocking ELISA (bELISA). The optimized amount of coated PCV2-Cap and dilution of mAb-1E7 were selected when the OD_450nm_ value was approximately 1.0 in the indirect ELISA. After the conditions mentioned above of bELISA are determined, the following procedures are performed. First, the ELISA plates were coated using the amount of PCV2-Cap (100, 200, and 400 ng/well) in PBS buffer at 4°C overnight. Second, after washing three times with PBS'T, the plates were blocked with blocking buffer (200 µL/well) at room temperature for 1 h. Meanwhile, the amount of mAb-1E7 at a volume of 100 µL was incubated for 1 h at 37°C with 100 µL of serially diluted nanobodies (1, 0.1, and 0.01 µg/well). After incubation, the ELISA plates were added to the mixture and incubated for 1 h at 37°C. Then, the plates were added with Peroxidase-AffiniPure goat anti-mouse IgG antibody and incubated for 1 h at 37°C. After being washed again, the plates were added with TMB (100 µL/well). Finally, after the reactions were stopped with 3 M H_2_SO_4_ (50 µL/well), the OD_450nm_ values were read with an automatic microplate reader.

### Affinity measurements

SPR experiments were performed on a Biacore 8K instrument. The proteins were purified to homogeneity by affinity chromatography. The mAb-1E7 was immobilized on a Protein G chip by standard amine coupling. The PCV2-Cap or Nb61 was injected over the chip at 30  µL/min in buffer (10 mM HEPES pH 7.4, 150 mM NaCl, 3 mM EDTA, 0.05% Tween 20) at concentrations ranging from 1.5625 to 25  nM. The sensor surface was regenerated between cycles with two 60 s injections of 10 mM glycine pH 1.5. Each sensorgram was corrected for nonspecific binding by subtracting the signal from the negative control flow cell. The sensorgrams were analyzed using Biacore 8K Evaluation software (Biacore) by applying a binding model to obtain kinetic affinity constants.

### Immunization of mice to generate antibodies against nanobodies

BABL/c mice were purchased from Chengdu Dossy Experimental Animals Co., LTD, China. Thirty-six female BALB/c mice (6-week-old) were randomly divided into six groups and immunized with the nanobodies (Table S2) using a dose of 100 µg per mouse. This immunization was repeated 2 weeks later. Sera were collected from the immunized mice 2 weeks after the fourth immunization. Anti-PCV2 antibodies were determined in sera with an indirect ELISA, using the recombinant PCV2-Cap protein (400 ng/well) as a coating antigen.

### Animal experiments

Twenty-five female BABL/c mice (6-week-old) were randomly assigned into five groups and used to design for immunization and virus-challenging experiments (Table S3). The negative control (PBS/MEM) and positive control (PBS/PCV2b) groups were immunized with 0.1 mL PBS. The other three groups were immunized with 100 µg Nb61, 200 µg Nb61, and 50 µg PCV2-Cap, respectively, and then were boosted with the same proteins 2 weeks later. Immunization was performed by intraperitoneal injection. Four weeks after primary immunization, all mice except the negative control group were challenged with PCV2b and then monitored for 28 days. Serum samples were collected on days 0, 7, 14, 21, 28, 42, and 56 after the first immunization to detect antibodies to PCV2b. Serum samples of mice were tested with the PCV2 ELISA kit (JNT, Beijing, China). Samples were considered positive if the calculated sample-to-positive (S/P) ratio was ≥0.4. Following the viral challenge, mice were monitored for 28  days and euthanized. Serum and lymph node samples were obtained for viral load analyses.

Piglets aged 3 weeks (15) were obtained from a pig farm and negative for PCV2 antigen and antibody by detecting qPCR and commercial ELISA kit (JNT, Beijing, China). Fifteen piglets were randomly divided into five groups (*n* = 3). Details of the piglets’ groupings are provided in Table S4. The groups were unimmunized with protein (PBS/MEM and PBS/PCV2b group) or were immunized with 50 µg Nb61, 500 µg Nb61, and 50 µg PCV2-Cap and then were boosted with the same proteins 2 weeks later. Immunization was performed by intramuscular injection. Four weeks after primary immunization, piglets were challenged with PCV2b and then monitored for 28 days. Serum samples were collected on days 0, 7, 14, 21, 28, 42, and 56 after the first immunization to detect antibodies to PCV2b. Serum samples of pigs were tested with the PCV2 ELISA kit (JNT, Beijing, China). Samples were considered positive if the calculated sample-to-positive (S/P) ratio was ≥0.4. Following the viral challenge, piglets were monitored for 28 days and euthanized. Serum and lymph node samples were also obtained for viral load analyses.

### Immunofluorescence assay

To determine mAb-1E7 binding to PCV2-Cap in the PCV2-infected PK‐15 cells, PK‐15 cells were infected with 100 TCID_50_ of PCV2b SH strain. Then, the infected PK‐15 cells were fixed with 4% Paraformaldehyde (Sigma-Aldrich) for 15 min at 37°C and permeated with 0.25% Triton X-100 (Sigma-Aldrich) for 5 min. After washing three times with PBS, the fixed cells were blocked by 1% BSA for 30 min at room temperature. After being washed again, the mAb-1E7 (1 µg/mL) was added and incubated for 1 h at room temperature. Then, Alexa flour 488 conjugated-goat anti-mouse IgG (H + L) as a secondary antibody was incubated for 1 h at room temperature. Finally, cells were stained with FluoroshieldTM with DAPI (Sigma-Aldrich) and observed with a Leica SP8 confocal system (Leica, Wetzlar, Germany). All the images were captured and processed using Leica Application Suite X (Version 1.0. Leica Microsystems).

### Virus neutralization assays

A viral neutralization assay was conducted as previously described (70). The PCV2b (200 TCID50, 100 µL) was initially incubated with 100  µL of serially diluted mAbs or positive sera for anti-PCV2b antibodies for 1 h at 37°C. After incubation, this mixture was added to PK‐15 cells in a 96-well plate. After incubating for 2 h at 37°C, the cells were washed twice with PBS and added with a fresh medium. Then, after 3 days, the treated cells were tested by IFA to quantify viral infection.

### Quantitative real-time PCR

PCV2 DNA was extracted using EasyPure Viral DNA/RNA Kit (TransGen Biotech, Beijing, China) from the virus-infected cells, sera, and lymph nodes of infected pigs and mice. Then, the copy numbers of PCV2 DNA were quantified according to the manufacturer’s instructions with an Applied Biosystem StepOnePlus Real-Time PCR System (Applied Biosystems, CA, United States). Primers (q-PCR-F/R, Table S7), RealStar Green Power Mixture (GenStar), DNA templates, and ddH_2_O were mixed in a PCR tube up to 20 µL. Then, the PCR amplification was performed at 95°C for 10  min, followed by 30 cycles of amplification at 95°C for 15 s and 60°C for 30 s.

### Peptide synthesis

To identify mAbs recognized the epitopes, 61 overlapping peptides of 15 amino acids (containing an offset of 4 aa) were synthesized by GenScript Biotech (Nanjing, China; Table S6) according to the amino acid sequences of PCV2-Cap (PCV2b SH strain) and 28 overlapping peptides of 15 amino acids were synthesized based on the sequences of Nb61 (Table S5). The purity of all synthetic peptides is more than 95%.

For ELISA assays to identify the epitope recognized by the mAb-1E7/mAb-3G4, the ELISA plates were coated with the peptides (1 µg/well). Then, mAb-1E7/mAb-3G4 was added to the plates, followed by the Peroxidase-AffiniPure goat anti-mouse IgG antibody. After washing, the plates were added with TMB (100 µL/well). Finally, after the reactions were stopped with 3 M H_2_SO_4_ (50 µL/well), the OD_450nm_ values were read with an automatic microplate reader.

### Dot immunoblotting

To further identify the epitope recognized by the mAb-1E7/mAb-3G4, the 3 µg of synthetic peptide was spotted onto nitrocellulose (NC) membranes and dried at room temperature. Then, the membranes were blocked with 5% skimmed milk in PBS'T and incubated with mAb-1E7/mAb-3G4 at 37°C for 1 h. After washing five times with PBS'T, the membranes were incubated with the Peroxidase-AffiniPure goat anti-mouse IgG antibody and developed with an ECL reagent.

### Production of monoclonal antibody against Nb61

Four 6-week-old female BALB/c mice were immunized intraperitoneally with Nb61, using a dose of 50 µg per mouse. This immunization was repeated 2 weeks later, and after a further 2 weeks, mice were administered 100 µg Nb61 intraperitoneally. The mice were euthanized 3 days later, and 50% PEG was used to fuse the spleen cells with SP2/0 cells. After 12 days of selection in the HAT medium, hybridoma colonies were tested for Ab3 production and serially diluted to obtain single-cell clones. Ab3 was purified by Protein G Resin (GenScript, China) for further characterization.

### Biological information analysis

The spatial distribution of epitopes was visualized by mapping the epitope on PCV2-Cap and Nb61 using the PyMOL. To further precisely define the key amino acid for PCV2-Cap and Nb61 binding to mAb-1E7 or mAb-3G4, the 3D structures of homology modeling for PCV2-Cap, Nb61, mAb-1E7, and mAb-3G4 were generated by the amino acid sequences submitted to the AlphaFold2 server. Then, the docking models of interactions were developed using the docking program on the server ZDOCK (zdock.umassmed.edu). Binding sites were analyzed using PyMOL.

### Statistical analysis

Statistical analysis was performed using GraphPad Prism version 8.0. Statistical significance between the two groups was analyzed using an unpaired Student’s *t*-test, and differences between three or more groups were compared with a control group using a one-way ANOVA. Asterisks indicate the statistical significance: NS, no significance; *P < 0.05, **P < 0.01, ***P < 0.001. *P* < 0.05 was considered to indicate a statistically significant difference.

## Data Availability

All study data are included in the article and/or supplemental material.

## SUPPLEMENTAL MATERIAL

Supplemental material jvi.01650-23-s0001.doc Figures S1 to S7 and Tables S1 to S7.

ASM does not own the copyrights to Supplemental Material that may be linked to, or accessed through, an article. The authors have granted ASM a non-exclusive, world-wide license to publish the Supplemental Material files. Please contact the corresponding author directly for reuse.
