# Supplementary material for: A novel strategy for an anti-idiotype vaccine: nanobody mimicking neutralization epitope of porcine circovirus type 2
Source: J Virol. 2024 Jan 25;98(2):e01650-23. doi: 10.1128/jvi.01650-23 (PMC10878242; doi:10.1128/jvi.01650-23)
Supplement: Supplemental material — Figures S1 to S7 and Tables S1 to S7. [file jvi.01650-23-s0001.doc]

**Supporting Information for**

**A novel strategy for an anti-idiotype vaccine: nanobody mimicking neutralization epitope of porcine circovirus type 2**

Yingying Deng^1, 2, 3†^, Yamin Sheng^1, 2, 3†^, Guixi Zhang^1, 2, 3^, Yani Sun^1, 2, 3^, Lei Wang^1, 2, 3^, Pinpin Ji^1, 2, 3^, Jiahong Zhu^1, 2, 3^, Gang Wang^4^, Baoyuan Liu^1, 2, 3^, En-Min Zhou^1^, Xuehui Cai^5^, Yabin Tu^5^, Julian A. Hiscox^6^, James P. Stewart^6^, Yang Mu^1, 2, 3*^, Qin Zhao^1, 2, 3*^

^1^Department of Preventive Veterinary Medicine, College of Veterinary Medicine, Northwest A&F University, Yangling, Shannxi 712100, China

^2^Engineering Research Center of Efficient New Vaccines for Animals, Universities of Shaanxi Province and Ministry of Education, Yangling, China

^3^Key Laboratory of Ruminant Disease Prevention and Control (West), Ministry of Agriculture and Rural Affairs, Yangling, China

^4^College of Veterinary Medicine, Shandong Agricultural University, Tai'an 271002, China

^5^Harbin Veterinary Research Institute, Chinese Academy of Agricultural Sciences, Harbin 150069, China

^6^Department of Infection Biology and Microbiomes, Institute of Infection, Veterinary and Ecological Sciences, University of Liverpool, Liverpool L3 5RF, United Kingdom

^*^Correspondences: muyang@nwafu.edu.cn (Y.M.), qinzhao_2004@nwsuaf.edu.cn (Q.Z.)

^†^These authors contributed equally to this work.


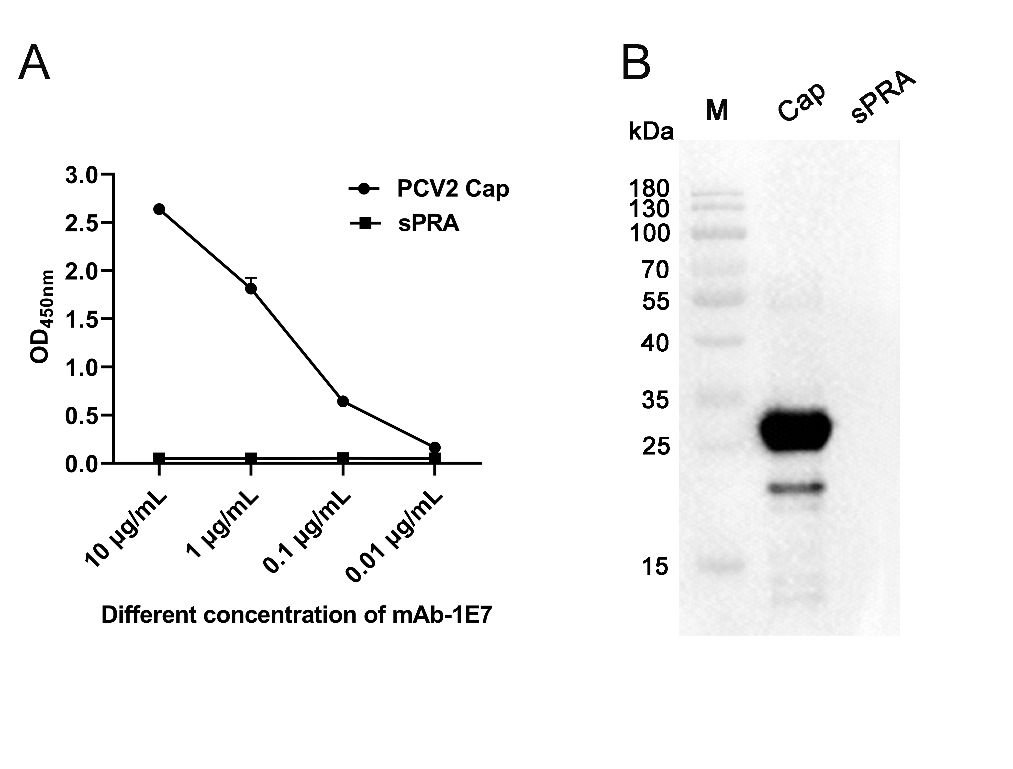


**Fig. S1. mAb-1E7 recognized the PCV2 Cap.** (A) ELISA and (B) Western blot analysis of mAb-1E7 binding to PCV2-Cap expressed with prokaryotic system. Different concentrations of mAb-1E7 interacting with PCV2-Cap. sPRA were used as negative protein controls, which has the same expression system as PCV2-Cap.


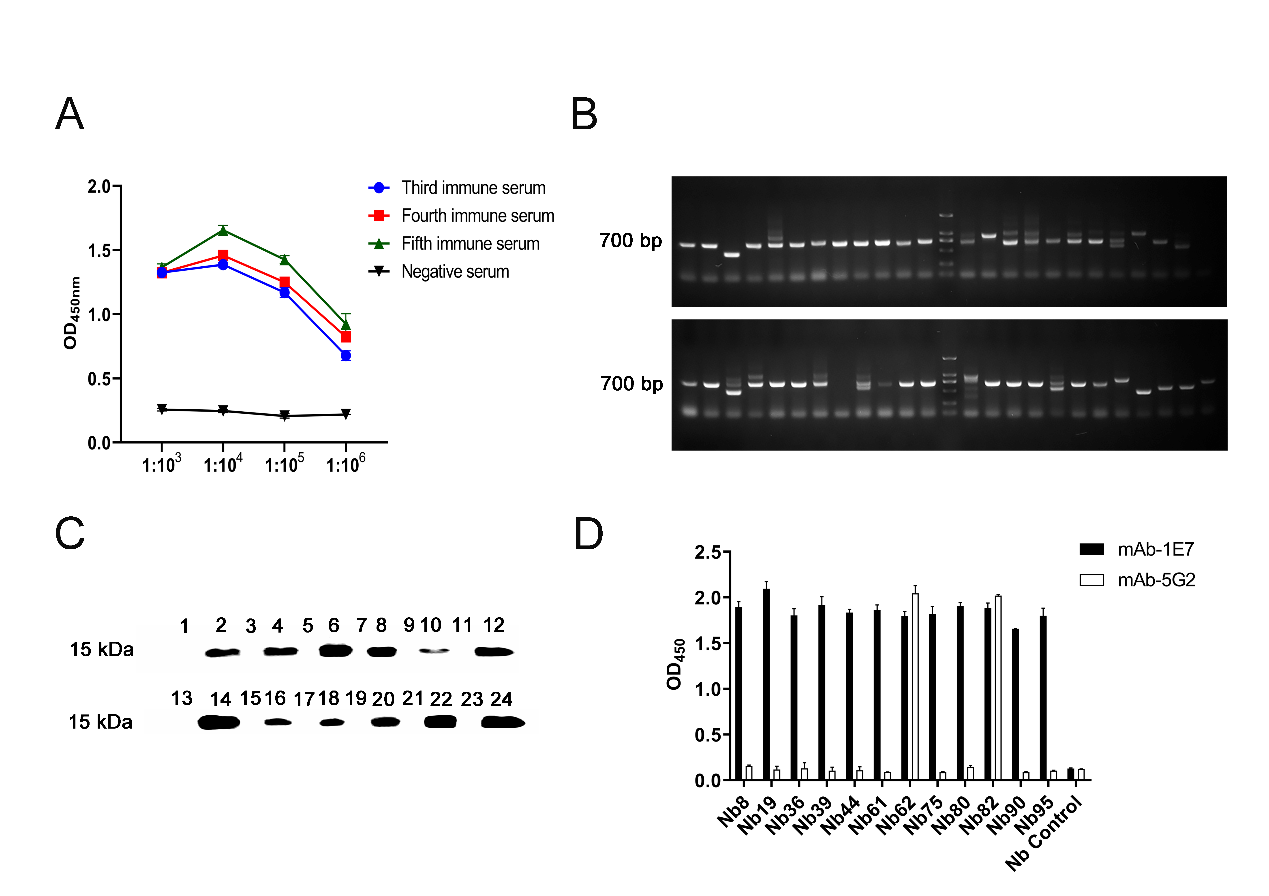


**Fig. S2. Screening of nanobodies against mAb-1E7 and production of nanobodies with the Pichia pastoris expression system.** (A) Titers of specific antibodies against mAb-1E7 in the immunized camel’s sera. (B) A total of 48 clones were randomly picked to estimate the correct insertion rate by PCR. The size of PCR products was approximately 700 bp. (C) Expression of the 12 specific nanobodies against mAb-1E7 by Western blot analysis. M: Marker; lanes 1, 3, 5, 7, 9, 11, 13, 15, 17, 19, 21, 23: Un-induced supernatant of Nb8, Nb19, Nb36, Nb39, Nb44, Nb61, Nb62, Nb75, Nb80, Nb82, Nb90 and Nb95 respectively; lanes 2, 4, 6, 8, 10, 12, 14, 16, 18, 20, 22, 24: supernatants of induced Nb8, Nb19, Nb36, Nb39, Nb44, Nb61, Nb62, Nb75, Nb80, Nb82, Nb90 and Nb95, respectively. (D) Detection of the 12 recombinant nanobodies specifically binding to the mAb-1E7 by indirect ELISA.


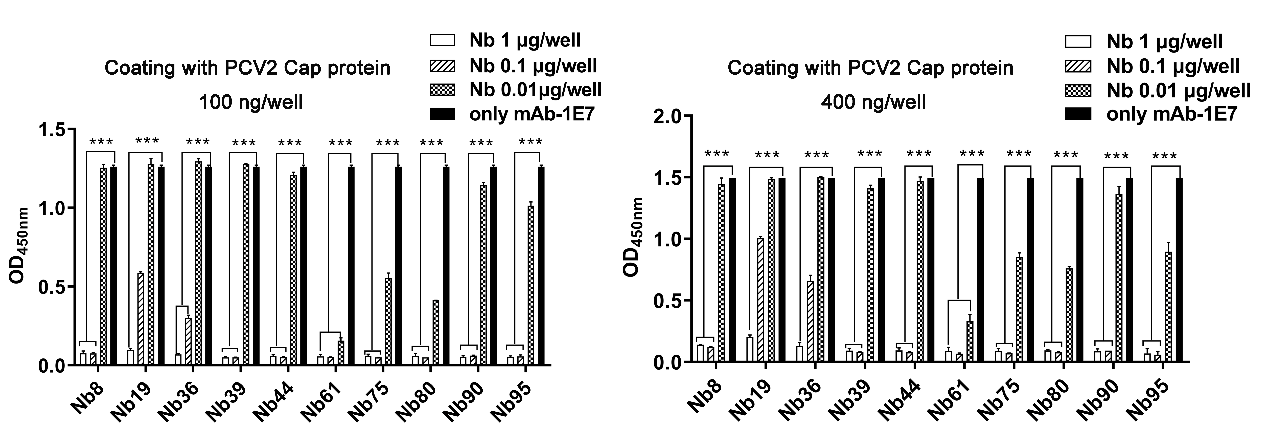


**Fig. S3.** Determination of nanobodies blocking PCV2-Cap (coating with 200/400 ng/well) reaction with mAb-1E7 by blocking ELISA.


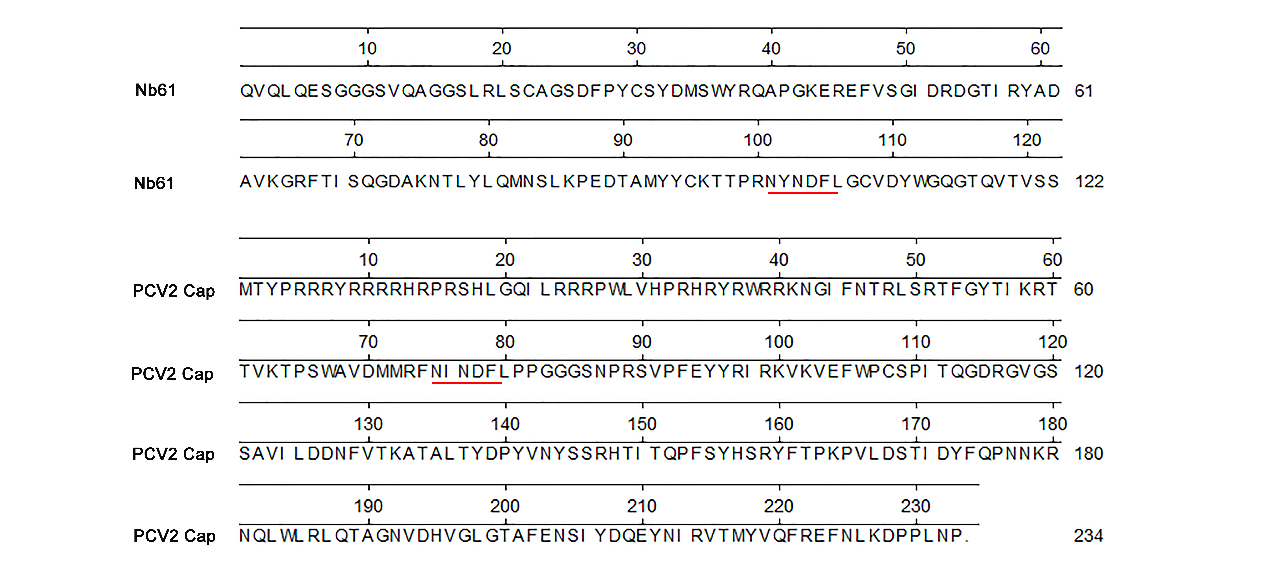


**Fig. S4.** Comparisons of the amino acid sequences between Nb61 and PCV2-Cap.


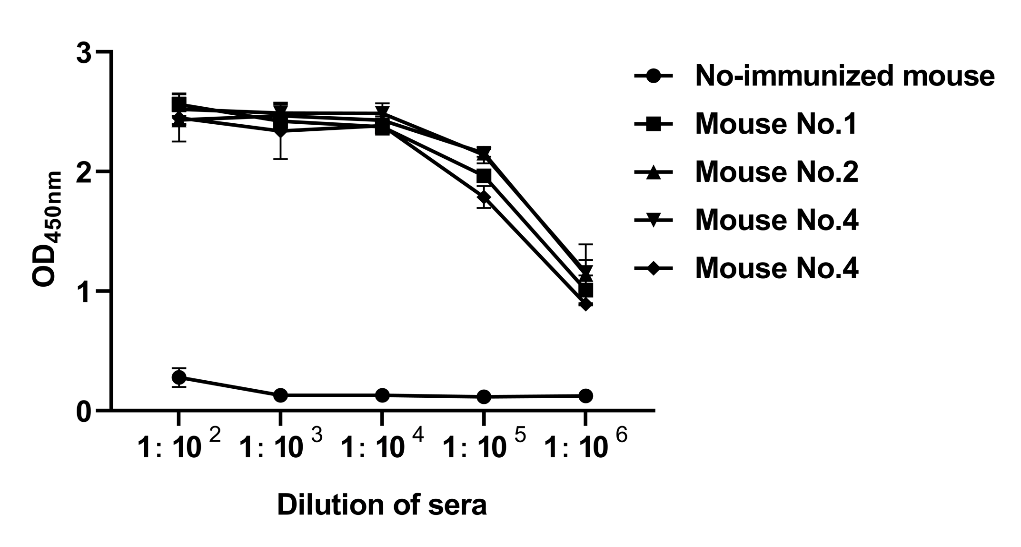


**Fig. S5.** Titers of specific antibody against Nb61 in the immunized mice sera.


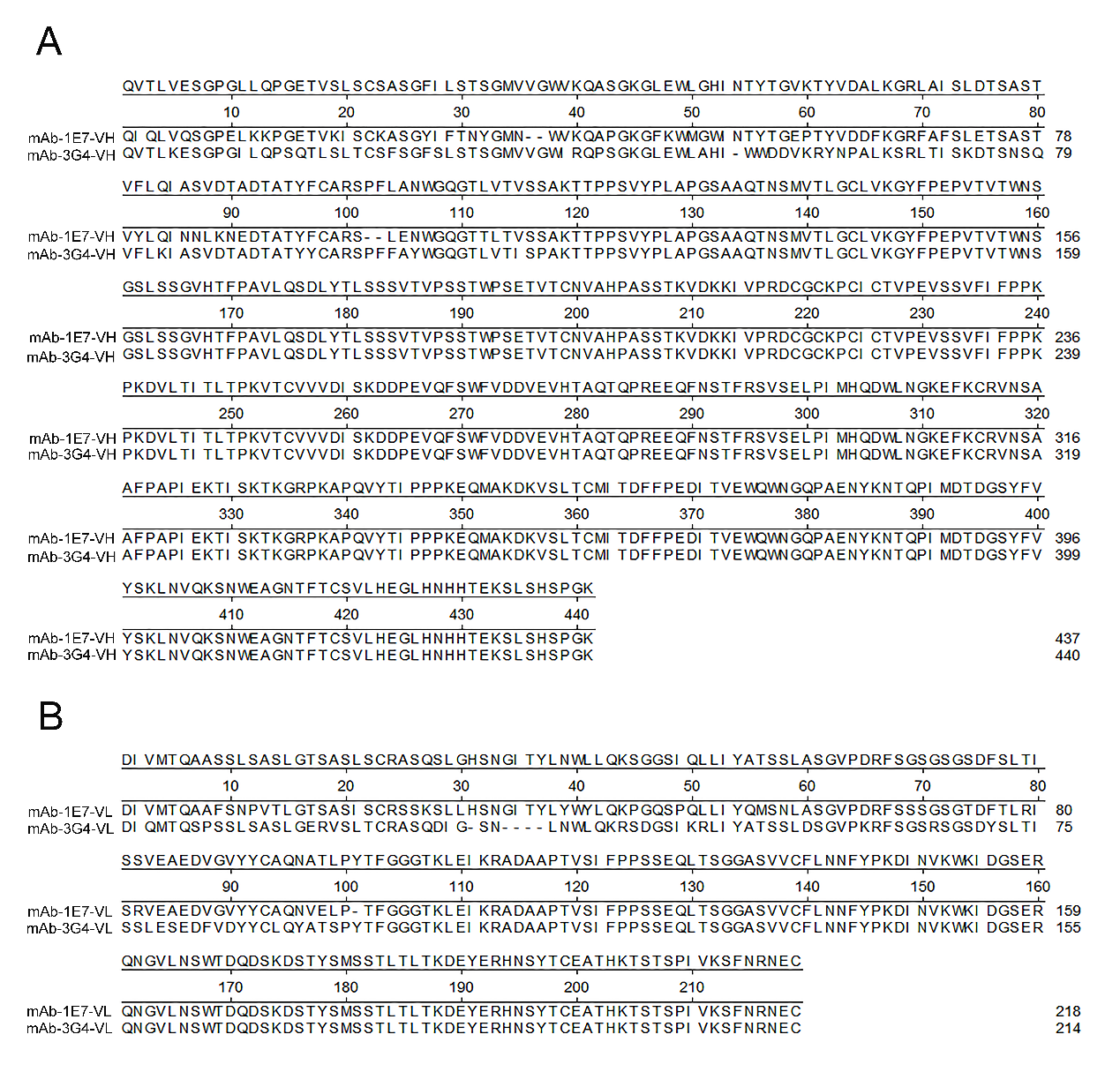


**Fig. S6.** Amino acids alignments of variable region of heavy (A) and light (B) chains of mAb-1E7 and mAb-3G4.


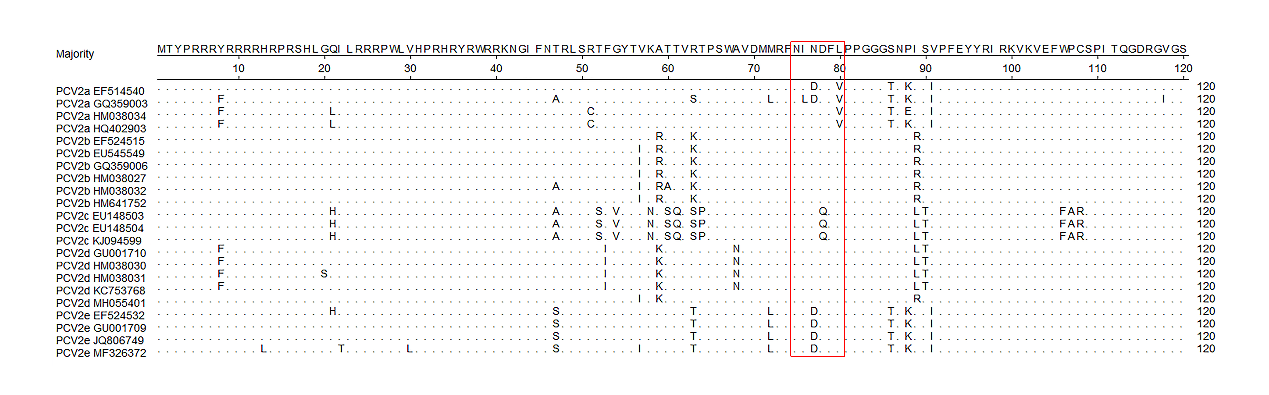


**Fig. S7.** Partial amino acid sequences alignments of different subtypes PCV2-Cap proteins.

**Table S1 Enrichment of phage particles during the 3 cycles**

| Round of screening | Input (Pfu/well) | P output (Pfu/well) | N output (Pfu/well) | Enrichment  (P/N) |
| --- | --- | --- | --- | --- |
| 1 | 5×10^10^ | 8×10^4^ | 2×10^4^ | 4 |
| 2 | 5×10^10^ | 9×10^5^ | 6×10^3^ | 1.5×10^2^ |
| 3 | 5×10^10^ | 1.5×10^6^ | 2×10^3^ | 7.5×10^2^ |

**Table S2 BALB/c mice were immunized with different nanobodies agaisnt mAb-1E7**

| Group name | Immunogen |
| --- | --- |
| 1 | Nb39 and Nb61 |
| 2 | Nb8 and Nb19 |
| 3 | Nb36 and Nb44 |
| 4 | Nb75 and Nb80 |
| 5 | Nb90 and Nb95 |
| 6 | Nb control |

**Table S3 Experimental design of mice immunized with Nb61 or PCV2-Cap following challenged with PCV2b**

| Group | Immunogen | Dose | Challenge isolate | Challenge dose (TCID_50_/ml) |
| --- | --- | --- | --- | --- |
| PBS/MEM | PBS | 0.1ml | / | / |
| PBS/PCV2b | PBS | 0.1ml | PCV2b | 1×10^6^/0.1ml |
| 100µg Nb61/PCV2b | Nb61 | 100µg /0.1ml | PCV2b | 1×10^6^/0.1ml |
| 200µg Nb61/PCV2b | Nb61 | 200µg /0.1ml | PCV2b | 1×10^6^/0.1ml |
| 50µg Cap/PCV2b | Cap | 50µg /0.1ml | PCV2b | 1×10^6^/0.1ml |

**Table S4 Experimental design of piglets immunized Nb61 or PCV2-Cap following challenged with PCV2b**

| Group | Immunogen | Dose | Challenge isolate | Challenge dose (TCID_50_/ml) |
| --- | --- | --- | --- | --- |
| PBS/MEM | PBS | 1ml | / | / |
| PBS/PCV2b | PBS | 1ml | PCV2b | 1×10^6^/2ml |
| 50µg Nb61/PCV2b | Nb61 | 50µg /1ml | PCV2b | 1×10^6^/2ml |
| 500µg Nb61/PCV2b | Nb61 | 500µg /1ml | PCV2b | 1×10^6^/2ml |
| 50µg Cap/PCV2b | Cap | 50µg /1ml | PCV2b | 1×10^6^/2ml |

**Table S5 Peptides of anti-idiotypic nanobody (Nb61)**

| Peptide  position | amino acid | Peptide  position | amino acid |
| --- | --- | --- | --- |

| 1-15 | QVQLQESGGGSVQAG | 57-71 | IRYADAVKGRFTISQ |
| --- | --- | --- | --- |
| 5-19 | QESGGGSVQAGGSLR | 61-75 | DAVKGRFTISQGDAK |
| 9-23 | GGSVQAGGSLRLSCA | 65-79 | GRFTISQGDAKNTLY |
| 13-27 | QAGGSLRLSCAGSDF | 69-83 | ISQGDAKNTLYLQMN |
| 17-31 | SLRLSCAGSDFPYCS | 73-87 | DAKNTLYLQMNSLKP |
| 21-35 | SCAGSDFPYCSYDMS | 77-91 | TLYLQMNSLKPEDTA |
| 25-39 | SDFPYCSYDMSWYRQ | 81-95 | QMNSLKPEDTAMYYC |
| 29-43 | YCSYDMSWYRQAPGK | 85-99 | LKPEDTAMYYCKTTP |
| 33-47 | DMSWYRQAPGKEREF | 89-103 | DTAMYYCKTTPRNYN |
| 37-51 | YRQAPGKEREFVSGI | **93-107** | YYCKTTPR**NYNDFL**G |
| 41-55 | PGKEREFVSGIDRDG | **97-111** | TTPR**NYNDFL**GCVDY |
| 45-59 | REFVSGIDRDGTIRY | **101-115** | **NYNDFL**GCVDYWGQG |
| 49-63 | SGIDRDGTIRYADAV | 105-119 | FLGCVDYWGQGTQVT |
| 53-67 | RDGTIRYADAVKGRF | 109-122 | VDYWGQGTQVTVSS |

**Table** **S6 Peptides of PCV2-Cap protein**

| **Peptide**  **position** | **amino acid** | **Peptide**  **position** | **amino acid** |
| --- | --- | --- | --- |
| 1-15 | MTYPRRRYRRRRHRP | 101-115 | VKVEFWPCSPITQGD |
| 5-19 | RRRYRRRRHRPRSHL | 105-119 | FWPCSPITQGDRGVG |
| 9-23 | RRRRHRPRSHLGQIL | 109-123 | SPITQGDRGVGSSAV |
| 13-27 | HRPRSHLGQILRRRP | 113-127 | QGDRGVGSSAVILDD |
| 17-31 | SHLGQILRRRPWLVH | 117-131 | GVGSSAVILDDNFVT |
| 21-35 | QILRRRPWLVHPRHR | 121-135 | SAVILDDNFVTKATA |
| 25-39 | RRPWLVHPRHRYRWR | 125-139 | LDDNFVTKATALTYD |
| 29-43 | LVHPRHRYRWRRKNG | 129-143 | FVTKATALTYDPYVN |
| 33-47 | RHRYRWRRKNGIFNT | 133-147 | ATALTYDPYVNYSSR |
| 37-51 | RWRRKNGIFNTRLSR | 137-151 | TYDPYVNYSSRHTIT |
| 41-55 | KNGIFNTRLSRTFGY | 141-155 | YVNYSSRHTITQPFS |
| 45-59 | FNTRLSRTFGYTIKR | 145-159 | SSRHTITQPFSYHSR |
| 49-63 | LSRTFGYTIKRTTVK | 149-163 | TITQPFSYHSRYFTP |
| 53-67 | FGYTIKRTTVKTPSW | 153-167 | PFSYHSRYFTPKPVL |
| 57-71 | IKRTTVKTPSWAVDM | 157-171 | HSRYFTPKPVLDSTI |
| 61-75 | TVKTPSWAVDMMRFN | 161-175 | FTPKPVLDSTIDYFQ |
| 65-79 | PSWAVDMMRFNINDF | 165-179 | PVLDSTIDYFQPNNK |
| **69-83** | VDMMRF**NINDFL**PPG | 169-183 | STIDYFQPNNKRNQL |
| **73-87** | RF**NINDFL**PPGGGSN | 173-187 | YFQPNNKRNQLWLRL |
| **74-88** | F**NINDFL**PPGGGSNP | 177-191 | NNKRNQLWLRLQTAG |
| **75-89** | **NINDFL**PPGGGSNPR | 181-195 | NQLWLRLQTAGNVDH |
| **76-90** | **INDFL**PPGGGSNPRS | 185-199 | LRLQTAGNVDHVGLG |
| **77-91** | **NDFL**PPGGGSNPRSV | 189-203 | TAGNVDHVGLGTAFE |
| **78-92** | **DFL**PPGGGSNPRSVP | 193-207 | VDHVGLGTAFENSIY |
| **79-93** | **FL**PPGGGSNPRSVPF | 197-211 | GLGTAFENSIYDQEY |
| **80-94** | **L**PPGGGSNPRSVPFE | 201-215 | AFENSIYDQEYNIRV |
| 81-95 | PPGGGSNPRSVPFEY | 205-219 | SIYDQEYNIRVTMYV |
| 85-99 | GSNPRSVPFEYYRIR | 209-223 | QEYNIRVTMYVQFRE |
| 89-103 | RSVPRSVPFEYYRIR | 213-227 | IRVTMYVQFREFNLK |
| 93-107 | FEYYRIRKVKVEFWP | 217-231 | MYVQFREFNLKDPPL |
| 97-111 | RIRKVKVEFWPCSPI |  |  |

**Table S7. Primer pairs used in the study**

| **Primer Names** | **Sequences (5’-3’)** |
| --- | --- |
| CALL001 | GTCCTGGCTGCTCTTCTACAAGG |
| CALL002 | GGTACGTGCTGTTGAACTGTTCC |
| VHH-FOR | GATGTGCAGCTGCAGGAGTCTGGRGGAGG |
| VHH-REV | CTAGTGCGGCCGCTGAGGAGACGGTGACCTGGGT |
| p5E-FOR | AATACGCAAACCGCCTCTCC |
| q-pcr-F | GAGTGGGCTCCAGTGCTGTTATT |
| q-pcr-R  pPICZαA-Nbs-F  pPICZα A-Nbs-R | GGAGAAGGGCTGGGTTATGGTAT CCGCTCGAGAAAAGACAGGTCCAACTGCAG  GCTCTAGACGTGAGGAGACGGTGACCT |
